# Stochastic Resonance Modulates Neural Synchronization within and between Cortical Sources

**DOI:** 10.1371/journal.pone.0014371

**Published:** 2010-12-16

**Authors:** Lawrence M. Ward, Shannon E. MacLean, Aaron Kirschner

**Affiliations:** 1 Department of Psychology, University of British Columbia, Vancouver, Canada; 2 The Brain Research Centre, University of British Columbia, Vancouver, Canada; Cuban Neuroscience Center, Cuba

## Abstract

Neural synchronization is a mechanism whereby functionally specific brain regions establish transient networks for perception, cognition, and action. Direct addition of weak noise (fast random fluctuations) to various neural systems enhances synchronization through the mechanism of stochastic resonance (SR). Moreover, SR also occurs in human perception, cognition, and action. Perception, cognition, and action are closely correlated with, and may depend upon, synchronized oscillations within specialized brain networks. We tested the hypothesis that SR-mediated neural synchronization occurs within and between functionally relevant brain areas and thus could be responsible for behavioral SR. We measured the 40-Hz transient response of the human auditory cortex to brief pure tones. This response arises when the ongoing, random-phase, 40-Hz activity of a group of tuned neurons in the auditory cortex becomes synchronized in response to the onset of an above-threshold sound at its “preferred” frequency. We presented a stream of near-threshold standard sounds in various levels of added broadband noise and measured subjects' 40-Hz response to the standards in a deviant-detection paradigm using high-density EEG. We used independent component analysis and dipole fitting to locate neural sources of the 40-Hz response in bilateral auditory cortex, left posterior cingulate cortex and left superior frontal gyrus. We found that added noise enhanced the 40-Hz response in all these areas. Moreover, added noise also increased the synchronization between these regions in alpha and gamma frequency bands both during and after the 40-Hz response. Our results demonstrate neural SR in several functionally specific brain regions, including areas not traditionally thought to contribute to the auditory 40-Hz transient response. In addition, we demonstrated SR in the synchronization between these brain regions. Thus, both intra- and inter-regional synchronization of neural activity are facilitated by the addition of moderate amounts of random noise. Because the noise levels in the brain fluctuate with arousal system activity, particularly across sleep-wake cycles, optimal neural noise levels, and thus SR, could be involved in optimizing the formation of task-relevant brain networks at several scales under normal conditions.

## Introduction

Neural synchronization is a putative mechanism whereby brain regions subserving specific functions communicate for the purpose of establishing transient networks that accomplish perception, cognition, and action [1]–[2]. It has been demonstrated, in model neurons, in slice preparations, and in whole brains, that neural synchronization is facilitated by the addition of optimal amounts of random fluctuations, or “noise,” to a neural network, whereas less than optimal amounts have less effect and larger than optimal amounts destroy synchronization [3]. This is one of a large class of such effects of noise on nonlinear systems that is called “stochastic resonance.” Moreover, it has also been demonstrated that SR occurs in human perception, cognition, and action as well as in various physiological preparations [4]. Several previous papers have speculated that SR-mediated neural synchronization is responsible for the behavioral SR effects [5]–[6]. In the present paper we provide new evidence consistent with this hypothesis. In addition, we describe several different modes of action of SR in the brain, both as enhancing local neural synchronization responsible for initial stimulus processing and indexed by local changes in spectral power in various frequency bands, as well as enhancing stochastic phase locking between distant brain regions cooperating in a network to manage processing of the effects of external stimuli. These results imply that SR-mediated neural synchronization is a general mechanism of brain functioning.

Synchronization as used here refers to the establishment and maintenance of a roughly constant difference between the oscillatory phases of weakly coupled oscillators through their mutual effects on each others' phases [7]. Physical synchronization was probably discovered by Huygens and has been an important topic in physics for many years. That it occurs in living systems has also been known for many years, and its study has been made easier by the introduction of models of synchronization in populations of weakly coupled phase oscillators [8]–[9]. Most recently, synchronization in complex systems, including chaotic systems, has been characterized [10]–[11]. Although several measures of synchronization have been introduced, particularly for studying chaotic systems, only a few have been widely adopted in neuroscience. In the present paper we use a measure closely related to the idea of roughly constant phase difference, but we acknowledge that more detailed descriptions of synchronization in the brain will be possible with the use of more sophisticated analyses [12].

Synchronous activity within neural networks in the gamma range of frequencies (30–50 Hz) is strongly associated with perception. This was first established robustly when Gray and Singer [13] showed that approximately 40-Hz oscillations were entrained and synchronized among cat primary visual cortical neurons that responded to the onset of a visual stimulus in their receptive fields. Among the many confirmatory results are those that established a similar response in human V1 [14] and in human A1 [15]. Synchronized gamma-frequency oscillations may be involved in binding together distributed neural representations of the external world [16], and may also play a role in producing perceptual awareness [16]–[17].

Importantly, noise can either enhance or destroy synchronization in networks of both model and real neurons [3]. Because of these effects stochastic resonance (SR) can occur in these networks. Synchronization-related SR is indicated whenever some optimal, non-zero, noise level leads to maximal synchronization of neural activity (spiking or oscillating dendritic currents) among elements of the network according to some appropriate metric. SR itself was discovered and named in physical systems. The first mention of SR seems to have been independently by Benzi, Sutara and Vulpiani [18] and by Nicolis [19] in describing subtle effects of solar variability on climate. A plethora of theoretical and experimental studies followed their work (reviewed many times but notably by Gammaitoni et al. [20]). One development highly relevant to neural systems was that of non-dynamical SR [21]. In this phenomenon, the all-important system non-linearity is simply a threshold such as that implemented in every neuron as the spiking threshold around -50 mV. This threshold can be “soft,” or gradual, as long as the transfer function results in areas of non-invertibility (or many-to-one mapping) between the below- and above-threshold regimes [22]. Such soft thresholds probably characterize those found in most living systems, including in neurons and in human psychophysical thresholds [23], as mathematically hard thresholds (i.e., a Heaviside function) are idealizations. Neural network SR was first described by Jung and Meyer-Kress [24] and has been studied extensively since then [25]. Thus, because SR can affect neural synchronization, it could play an important role in the brain implementation of perceptual and cognitive processes and even in the generation of primary awareness.

The first direct evidence that SR might operate in the human brain, to our knowledge, was the study of Srebo and Malladi [26]. They found that the EEG steady-state visual evoked potential (VEP) to contrast-reversing (at 4 Hz) weak (20% contrast) square-wave gratings over occipital cortex was enhanced by the presence of a moderate level of flickering visual noise. Subsequently, Stufflebeam, Poeppel and Roberts [27] showed that the variability of the magnetoencephalographic (MEG) M100 response to 6 dB SL 200-Hz pure tones decreased in the presence of a moderate level of added noise.

Several more definitive studies have established more firmly the occurrence of SR in the human brain. Mori and Kai [28] found that 10 Hz (first harmonic of driving frequency) neural responses recorded by scalp electrodes placed over the occipital cortex were more strongly entrained by a sub-threshold 5-Hz flickering stimulus when intermediate amounts of random visual noise were added. Similarly, Manjarrez et al. [29] showed that the EEG signal-to-noise-ratio (SNR) near the driving frequency over somatosensory cortex to a 2.5 Hz mechanical stimulus applied to a finger was enhanced by an non-zero level of mechanical noise added to the stimulus, albeit a different level for different subjects (cf. [30]). Finally, Kitajo et al. [6] recorded the EEG while subjects performed a visual detection task in a design similar to that of Mori and Kai [28] and Kitajo et al. [5], with noise and stimulus presented to the two eyes separately so that the two were mixed in the brain rather than in the stimulus or at the receptor. They found that average phase locking statistics between all pairs of 19 electrodes distributed equidistantly over the scalp were maximal for the same, non-zero, noise condition at which performance peaked, and this occurred for all of the theta (4–7 Hz), alpha/beta (8–29 Hz) and gamma frequency bands. It is generally agreed that synchronous oscillations of dendritic currents among large numbers of cortical pyramidal neurons is the origin of the electrical potentials recorded by EEG [31]. Thus, we can conclude that the SR observed in these studies probably was caused by noise-induced changes in synchronization among cortical neurons.

Interestingly, an early study of masking of the 40-Hz auditory steady-state response (SSR) by broadband auditory noise reported the serendipitous discovery of a phenomenon that closely resembles SR [32]–[33]. Galambos and colleagues found that whereas high levels of noise reduced the magnitude of the EEG-recorded SSR, paradoxically a low level of ipsilateral noise, but not contralateral noise, actually enhanced the SSR relative to the no-noise condition. More recently, Tanaka, Kawakatsu and Nemoto [34] also found some intriguing evidence of SR in the auditory SSR using MEG.

Although these previous studies do show that SR seems to affect synchronization among neurons in the human brain, they do not provide information about exactly where these SR effects are occurring. The EEG or MEG scalp recordings analysed are comprised of a mixture of signals from many areas of the brain and are thus ambiguous as to the sources of these signals. One or more of the above-described studies are also are limited in generality to conditions of low frequency driving of neurons, at-rest, eyes-closed conditions, particular frequency bands, or single-sensor or averaged-over-sensors synchronization analysis. It is therefore not possible know on the basis of these previous studies whether SR affects intra-regional neural synchronization, inter-regional synchronization, or both, nor exactly which brain regions are displaying the SR effects.

We built upon this earlier work by implementing brain source analysis to obtain evidence of SR within and between localized brain regions. Because of the promising results already obtained for the auditory 40-Hz SSR we decided to study neural SR within this general domain. Because of the possibility of analyzing responses to single stimuli, however, we decided to examine the effects of added auditory noise on the closely related 40-Hz transient auditory response. This response also seemed to be a good choice because its neural etiology is fairly well understood and it is relevant to perception and attention [35] and thus to behavior.

The 40-Hz response of the human auditory cortex measured by EEG or by MEG is an index of neural synchronization that is directly related to the detection of environmental sounds. The transient 40-Hz response arises when the ongoing, random-phase, 40-Hz activity of tuned neurons in the auditory cortex is locked to the onset of a sound stimulus to which they respond, and a steady-state 40-Hz response (SSR) arises when the activity of responding neurons is phase-locked to a persistent 40-Hz modulation of a carrier sound [15], [36]. As the steady-state 40-Hz response recorded by EEG appears to arise from a summation of the potential oscillations generated by closely spaced transient 40-Hz responses, the two have often been treated as arising from the same neural sources [36]–[37]. Both transient and SSR responses are closely related to the behavioural threshold for detection of the sounds – that is, they appear at roughly the same sound level as that required for a behavioural response [38]–[40]. It has been suggested that the threshold for the 40-Hz response, particularly the SSR, can be used as a surrogate for the threshold for detection of sounds in those who cannot or will not make verbal or other behavioral responses in threshold tests [36], [41]. Although this works quite well for adults [41], the SSR at higher frequencies, 80 Hz in particular, is better for children and infants [41]–[42]. In our study we take advantage of this behavioral surrogate status in adults to present many more near-threshold stimuli than would be possible in a typical study that actually obtained behavioral responses to each stimulus (see Methods section).

**Figure 1:**
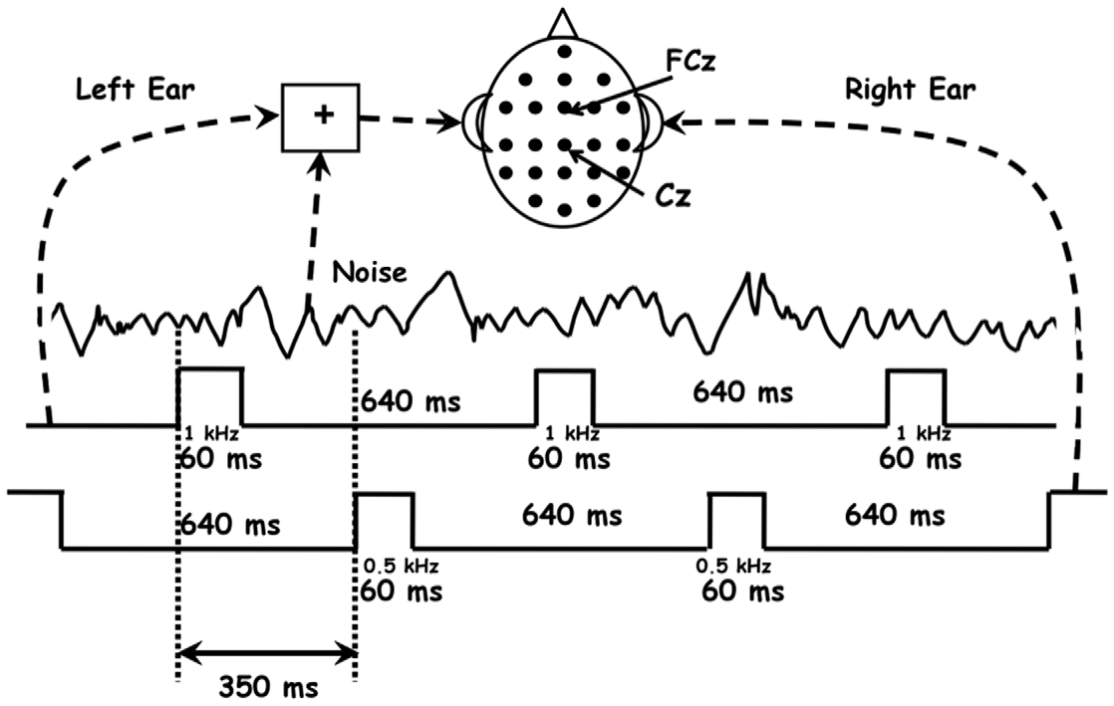
Experimental stimuli and procedure.

We adopted a paradigm similar to that used by Tiitinen et al. [35] who demonstrated the effect of attention on the transient 40-Hz response. We presented bilateral streams of peri-threshold sounds (1000-Hz at 5 dB SL to the left ear, termed “left standards,” and 500-Hz at 5 dB SL to the right ear, termed “right standards”) in the presence of various levels of added broadband acoustic noise (no-noise, -5, 0, 5, 10 and 20 dB SL to the left ear only) while recording 64-channel EEG (see Figure 1 for stimulus timing). The peri-threshold sounds were randomly mixed with occasional 20 dB SL intensity “deviants” (5%) at both frequencies, and subjects were required to push a button when they detected a deviant *in the left ear only*, so that they were attending to the stimulus stream in the left ear and ignoring that in the right ear. We localized the neural sources activated by this task that were common to most subjects using independent component analysis and subsequent single dipole fitting. We then measured the 40-Hz response to the peri-threshold standards, as well as inter-component synchronization, for selected independent components at each of the various levels of added acoustical noise in order to determine whether the noise would modulate synchronization in the brain, thus implicating SR. Although subjects did not respond behaviorally to the standard stimuli, the measurement of the 40-Hz transient response to those stimuli under the various noise conditions constitutes a surrogate for a behavioral response as described earlier, because the strength of the 40 Hz response is directly related to the probability of a behavioral response in a standard behavioral threshold task.

Independent component analysis (ICA) is a blind source separation method [43] that consists of decomposing the EEG time series, which consists of a linear mixture of signals from many sources, into a set of statistically independent signals called independent components (ICs) [44] prior to any dipole fitting procedure. ICA decomposition is useful as a method of artifact rejection to separate irrelevant physiological activities originating from ocular, muscular, and cardiac activity, as well as electrical interference (line noise), from relevant neural activity, based on the activity time courses, scalp maps, power spectra, and dipole locations of the ICs [45], thus increasing the signal-to-noise ratio of the experimental data. Another advantage of the ICA approach is that it requires no prior assumptions regarding the number or locations of active neural sources in a given paradigm (although of course there is often prior knowledge that constrains regions of interest, as in the present case). To determine which non-artifact ICs are task relevant, evidence is sought that some aspect of their activity was modulated by the task conditions. Furthermore, task-relevant ICs usually can be associated with single equivalent dipoles whose locations in the brain are highly similar across experimental subjects, significantly improving the usually poor spatial resolution of scalp EEG. Limitations to the data analysis approach taken in this study are discussed in the Discussion section and in relevant parts of the Methods section.

In the present study, EEG data analysis was comprised of the following steps, described in more detail in the Methods section: (1) decomposition of the continuous 64-channel EEG into 64 ICs for each participant separately; (2) selection and localization in the brain of the valid ICs (those with <15% residual variance in a single equivalent dipole fit localized to Talairach brain space - all other ICs were rejected as artifact or as uninterpretable); (3) division of the continuous record of each valid IC into epochs containing the peri-threshold left or right ear standards in each of the six noise conditions; (4) calculation of event-related spectral perturbations (ERSPs, change in spectral power from pre-stimulus baseline) for each IC in each subject's record; (5) cluster analysis of valid ICs to determine ICs common to most subjects; (6) summing ERSPs from cluster-selected ICs in a specified time-frequency window for analysis; and (7) calculation of cross-coherences (phase-locking statistics) for each subject between each pair of ICs for which there was an IC in each of two relevant clusters.

We expected that at least the primary auditory cortices would be active in this paradigm because the 40-Hz transient response has been localized there in previous studies [15], as well as possibly other areas in frontal and parietal cortex because these are usually active in any task requiring decisions based on perceptual input. We expected to see, in at least the auditory cortices and possibly other areas as well, the largest 40-Hz response, and also the most synchronization between active brain regions, for a non-zero level of added noise, demonstrating SR effects mediated by neural synchronization. We were uncertain which frequency bands might exhibit changes in long-range synchronization because theta (here 4–8 Hz), alpha (here 9–14 Hz), and gamma (30–50 Hz) bands all have been implicated in various ways in such phase locking [2], [46]–[49]. In general, synchronization at lower frequencies is expected for long-range interactions and that at higher frequencies is expected for local interactions, but there have been reports of functionally-related long-range synchronization in the gamma range [47]. The present study is thus somewhat exploratory regarding this aspect.

## Results

**Figure 2:**
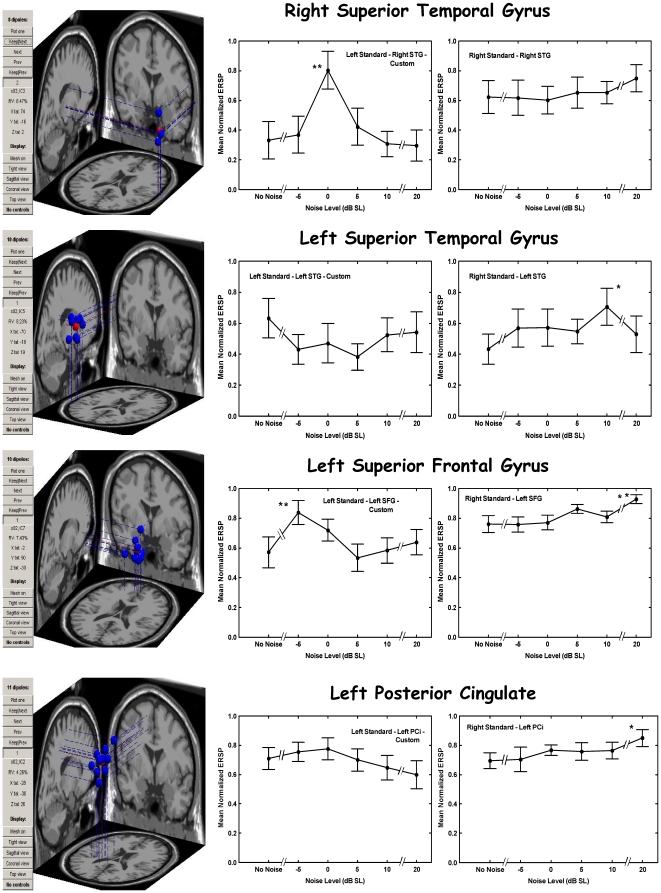
Neural source locations and normalized power ratios in those sources as a function of noise level. Left column depicts the locations of the individual sources in their clusters (blue dots) and the cluster centroids (red dots). The middle and right columns depict mean normalized power ratios plotted versus noise condition for the left standards (custom frequency window) and right standards (broad frequency window). Error bars indicate 1 standard error of the mean. *One asterisk next to a point means that the indicated maximum power ratio condition differs from no-noise condition by more than 2 standard errors. **Two asterisks next to a point means that the indicated maximum power ratio condition differs from no-noise condition by more than 2 standard errors *and* at *p*<0.05 by Dunnett's test in ANOVA setting. (See Figure S1 for results for left standard broad window and right standard custom window.)

Scalp topography maps of the centroids of all 20 clusters of the 200 valid ICs from our 10 subjects, and in particular of the four clusters we selected for intense scrutiny, uniformly indicated single equivalent dipole sources. The results reported here pertain to these four common clusters (at least 7 of 10 subjects represented by at least one IC), whose characteristics are described in Table 1 and whose equivalent dipole locations in the brain are illustrated in the left column of Figure 2. The remaining clusters were not analyzed further. A few subjects contributed multiple ICs to one or more of the common clusters. In these cases a single IC that showed the greatest SR effect in the power ratio (since the purpose of this study was to discover such effects) was selected and the others were discarded. In a single case for the left standard and a single case for the right standard no SR was evident among the several included ICs for a subject and in these two cases the IC showing the smallest departure from SR was chosen. The remainder of the few cases in which there was no SR effect evident occurred when only a single IC was available for that subject in that cluster and therefore that IC was included in the analysis.

**Table 1:** Cluster Properties.

Cluster Brain Region	# Subjects with valid IC	Total # of ICs	BA	Centroid Talairach x, y, z	Mean % RV from dipole fit	SD of RV
**R STG**	7/10	10	42	72, −11, 5	9.62	3.60
**L STG**	9/10	14	42	−71, −21, 11	8.28	4.44
**L SFG**	9/10	16	11	−4, 52, −22	8.47	3.11
**L PCi**	10/10	12	31	−24, −26, 39	6.63	3.96

**BA** Brodmann Area; **IC** independent component; **L** left; **PCi** posterior cingulate; **R** right; **RV** residual variance; **SD** standard deviation; **SFG** superior frontal gyrus; **STG** superior temporal gyrus.

Table 2 and Figure 2 summarize the analyses of spectral power ratios in the 40-Hz transient response time-frequency window derived from wavelet analyses of the IC time series (see Methods section for details of the calculations). Table 2 demonstrates that most subjects displayed an SR effect, in that the maximum power ratio in the 40-Hz response window (0–100 ms after onset of a standard) occurred for a non-zero noise level. This was the case for both types of frequency window determinations, but the custom analysis (frequency window chosen for individual subjects based on their 40-Hz response to the deviant stimuli) yielded better results for the left standards, whereas the broad analysis (30–50 Hz for all subjects) yielded better results for the right standards. In particular, all subjects with ICs localized to right superior temporal gyrus (R STG, n = 7) displayed SR for the left standard in the custom analysis, and all subjects with ICs localized to left superior temporal gyrus (L STG, n = 9) displayed SR for right standards in the broad analysis. These are the most likely regions to exhibit the transient 40-Hz auditory response, as these represent primary auditory sensory processing regions of cortex. Moreover, Table 1 indicates that most subjects also displayed SR in two non-sensory brain regions: left superior frontal gyrus (L SFG) and left posterior cingulate cortex (L PCi).

**Table 2:** Numbers of subjects with IC displaying SR by brain region.

Brain Region	Left Standard *Custom	Right Standard *Custom	Left Standard 30–50 Hz	Right Standard 30–50 Hz
**R STG**	7/7	6/7	6/7	6/7
**L STG**	6/9	8/9	7/9	9/9
**L SFG**	8/9	8/9	9/9	7/9
**L PCi**	9/10	9/10	10/10	9/10

**L** left; **SFG** superior frontal gyrus; **PCi** posterior cingulate; **R** right; **STG** superior temporal gyrus.

*Custom  =  frequency range determined for each subject separately from their frequency range for transient 40 Hz response to deviant stimuli (20 dB SL).

Figure 2 displays the mean normalized spectral power ratios for left standard (custom frequency window) and right standard (30–50 Hz frequency window) stimuli for each noise condition and for each of the four IC clusters localized to brain region. (See Figure S1 for the left standard 30–50 Hz window and right standard custom window data.) Statistically reliable SR is indicated in Figure 2 by two asterisks (or more weakly by a single asterisk) located near the error bar at a non-zero noise level, meaning that the normalized power ratio for that condition is significantly different from that of the no-noise condition. It can be seen that statistically reliable SR occurs only for the contralateral stimulus condition for L STG and R STG respectively.

Similar patterns of SR hold for left and right standards in the ICs localized to non-sensory brain regions, the L SFG and the L PCi. Again, the optimum noise level is higher for the right than for the left standards, presumably because of greater attenuation of noise-related activation within the relevant sensory pathways for the right standards (see Discussion). These results also demonstrate that the 40-Hz transient auditory response occurs more widely in the brain than just in the auditory cortex.

**Figure 3:**
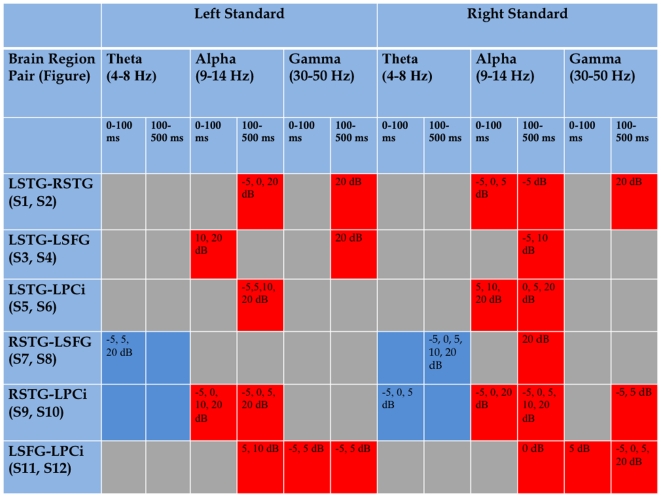
Significant differences in cross-coherence (phase locking values) between added-noise and no-noise conditions for indicated IC pairs. Red: Average phase-locking statistic over indicated time-frequency window for listed added-noise condition significantly different by non-parametric permutation-resampling test at *p*<0.001 from that in no-noise condition *and* phase locking significantly different from zero for several contiguous pixels in the added-noise condition by EEGLAB binomial test. Blue: Phase locking significantly different from zero for both no-noise and most or all added-noise conditions by EEGLAB binomial test with indicated added-noise conditions significantly different from the no-noise condition by permutation test. Gray: No significantly non-zero phase locking and/or no significant differences in phase locking between no-noise and an added-noise condition.

The involvement of several brain regions, particularly non-sensory ones, in the transient 40-Hz auditory response implies that there should be demonstrable interaction between these brain regions as information regarding the stimulus environment is passed among them. Figure 3 displays the results of the cross coherence (phase locking) analysis, which should be sensitive to at least some of the occasions on which such information passing is likely to be occurring, assuming that neuronal communication is facilitated by increased phase locking [50]. The colored entries in Figure 3 describe the results of a permutation-resampling non-parametric statistical comparison of added-noise conditions with the no-noise condition (see Methods section for analysis details and Figure 3 caption for meaning of the colored entries; detailed time-frequency plots that show phase-locking values in each condition that were significantly different from zero for the majority of subjects can be found in Figure S2). It is clear from Figure 3 that the brain regions that exhibit SR also exhibit significantly more phase-locking between them in alpha and gamma frequency bands, in at least one added-noise condition, than they do in the no-noise condition. This means that the added noise enhanced phase locking between relevant brain regions, often during the 0–100 ms interval in which we assessed the transient 40-Hz auditory response.

Also of interest in Figure 3 is the relationship between the LSFG and the RSTG, and between the LPCi and the RSTG. These pairs of regions appear to have been synchronized in the theta frequency band (blue colored squares) during nearly the entire experiment for nearly all noise conditions (see Figure S2). That is, because they were phase-locked during pretty much the entire duration of both right and left standard epochs, and because these epochs alternated, the phase locking must have been effectively continuous. The fact that this occurred for pretty much all noise conditions, including the no-noise condition, implies that it arose from the requirement to respond to the left ear deviant stimuli, presumably represented mostly by neural activity in the RSTG.

The central importance of the RSTG in the performance of the deviant detection task also appears in another way in the phase-locking analysis. In 11 of 14 instances of significant SR that involved the RSTG (RSTG-LSTG, RSTG-LSFG, RSTG-LPCi), noted in Figure 3, the lowest level of noise at which phase locking exceeded that in the no-noise condition was -5 dB. In contrast, in only 2 of 6 cases instances of increased phase locking involving LSTG and some other area was -5 dB the lowest noise level to show the effect. Thus, it would appear that the sensitivity to SR effects of the interaction of the RSTG with other areas was influenced by its role in processing of the deviant stimuli. Interestingly, this occurred both for left and right standards, possibly because of the establishment of the deviant-processing network via theta-band synchronization (see Discussion).

## Discussion

We have presented a set of results that establish the existence of SR effects both for the 40-Hz transient auditory response of the brain to near-threshold sound stimuli, implying effects on intra-regional neural synchronization, and for the synchronization of oscillations in several brain regions involved in processing the neural representations of these sounds. Such SR effects occur in all of the theta, alpha, and gamma frequency bands. These results raise a number of issues that require further discussion.

First, what roles could brain regions outside of auditory cortex be playing in processing of the stimuli in the present experiment? The L SFG has been implicated in diverse types of cortical information processing, including especially integration of sensory input with working memory and spatially oriented processing [51]. In the present case this could involve comparing the memory of a loudness deviant presented to the left ear with the current auditory input, similar to the frontal processing that occurs in other oddball tasks such as those that yield a mismatch negativity [52]. The PCi also performs several functions, including that of episodic memory retrieval [53], experiential but outward directed self-reflection such as thinking about duties and obligations [54], and attention allocation [55]. In the present context PCi activity could reflect the ongoing preoccupation with detecting, allocating attention to, and responding to the left-ear deviants. Both areas would be activated for any auditory stimulus that could be adequately represented in the brain and thus would require a decision to be made as to whether to respond to it (left ear deviant) or not (all others).

Second, it is striking that synchronization in the theta band has a more continuous and general character in this experiment, and is significantly non-zero even in the no-noise condition, whereas that in the alpha and gamma bands is more intermittent and tends to be significantly non-zero only in the added-noise conditions. This dissociation parallels that between stimuli that required a response (left-ear deviants) and those that did not (all others). It is consistent with the idea that the LPCi and the LSFG were continuously linked to the RSTG (but, interestingly, not to each other) via theta-band synchronization in order to make that discrimination, whereas the more intermittent linkages between other pairs of areas in the alpha and gamma bands represented transient communication relevant to the 40-Hz response elicited by deviants and standards alike (although much weaker and often absent for the standards).

Third, there is considerable asymmetry in the noise effects on the auditory cortex (see Figure 2). This is reasonable as there is significant hemispheric crossing in the auditory pathway from the cochlea through the brain stem nuclei to the primary auditory cortex, albeit not as complete as in the visual pathway from the retina. Moreover, the noise level at which the power ratio is significantly greater than that in the no-noise condition (thus exhibiting SR) is higher for the right standards (10 dB SL) than for the left standards (0 dB SL). This is also reasonable, as the noise would be integrated with the signal at the receptor for the left standard, where signal and noise were mixed physically, but only in the brain for the right standard. For the right standards, noise-related activation would be mixed with right-standard-stimulus-related activation either in subcortical auditory nuclei (possibly as early as the superior olive) or in auditory cortical regions (e.g., L STG or R STG; cf. [28]), although from the present results alone we cannot distinguish between these possibilities. Noise mixture in the brain would be expected to require a greater noise-to-signal ratio for effective SR according to a recent model because of attenuation of noise-related activation across multiple synapses [56].

Given the above discussion, one might also reasonably expect that primary sensory areas such as STG would display a maximum in the power ratio at a lower noise level than would areas more removed from the noise input, e.g., SFG and PCi. Although this is the case for right standards, there is an exception for left standards. The exception is the L SFG, which displays a maximum normalized power ratio for left standards at -5 dB of added noise whereas the R STG displays a maximum at 0 dB for the same stimuli. This result could indicate some additivity of correlated feed-forward noise. Because for left standards auditory noise was mixed with the signal before stimulus presentation, the random neural activity caused by this noise should remain correlated as it proceeded through various pathways in the brain, although this correlation would attenuate as activity on separate pathways was mixed with endogenous random neural activity. Nonetheless, it should still be the case that correlated feedforward noise would reach the L SFG both from the R STG (more strongly either because of a more direct pathway and/or because processing in this area was facilitated by the need to detect left deviants) and from the L STG (more weakly because attenuated by multiple synapses on a less direct pathway), in effect multiplying the nominal noise level by a factor greater than 1 and shifting the SR curve toward lower noise levels. In contrast, for right standards the exogenous noise would already be mixed with endogenous noise before it arrived at any brain area, and thus be much more weakly correlated across regions. Such weakly correlated noise would be expected to cancel rather than to add.

More generally, the issue arises as to what these data have to say about the way in which externally added noise (transformed into random neural activity) or endogenous neural noise is transmitted between and within brain regions in such cases. Although there is no general theory of this process available, the model of Lugo et al. [56] predicts that noise intensity must be greater for cross-modal SR than for uni-modal SR. This implies that noise energy is lost through multiple synaptic transfers occurring across sensory modalities. Moreover, it would be expected that addition of uncorrelated noise from multiple sources would result in lower noise output because the fluctuations would tend to cancel. Addition of correlated noises on the other hand would result in a greater noise output, depending on the correlation. It would also be expected that noise correlation would be destroyed by synaptic transmission, as additional noise sources would be added in at every neuron. So it should be the case that correlations between noise sources that have undergone multiple synaptic transmissions would be smaller than between those that have undergone fewer or no such transmissions. Finally, for signals that already have noise mixed into them, the neural activity caused by the signal+noise would be remain correlated, although with attenuation, as it progressed through various pathways. Wherever the pathways converged, some additivity, depending on the remaining correlation, would result. It might even be the case that site of convergence later in a feed-forward network might receive more noise from a given input than would a non-convergent site earlier in the network, as might have happened with the L SFG versus the R STG for the left standard stimuli, thus shifting the SR curve toward lower noise levels.

An interesting side issue also arises from the present results. Although confounded with whether the noise was added to the stimulus or mixed with it within the brain, it is apparent that attention did not abolish SR, as SR occurred for both left (attended ear) and right (unattended ear) standards. Indeed, attention might have contributed to the occurrence of SR at a generally lower noise level for attended left standards than for unattended right standards (although this cannot be determined from the present experimental design). Ward and Kitajo [57] had previously investigated the claim that attention to a stimulus served to attenuate any noise associated with that stimulus. They found evidence that although attention strongly affected detection of near-threshold visual stimuli, it only weakly affected SR as there was evidence of SR in both attended and unattended spatial locations. The present data reinforce their conclusion that attention does not attenuate noise for near threshold stimuli and also extend it to the auditory sensory domain. Rather, SR operates for weak stimuli in noise whether or not attention is being paid to them. This result also is consistent with the model of SR and attention proposed by Ward and Kitajo [57], in which a weighting function changes with stimulus strength, allowing SR for weak stimuli but enforcing attenuation of noise by attention for strong stimuli.

A final issue involves the limitations of the analysis techniques used in the present study. First, the extended infomax algorithm implemented in EEGLAB is a nonlinear blind source separation technique, and the information and other criteria used ensure that higher-order association statistics as well as second-order correlations are minimized [39], [40]. The technique has been extensively tested in many applications (see [58]–[59] for reviews) and has been shown to do an excellent job of recovering both radial and tangential neural sources [58]. Moreover, techniques such as ICA have been shown to help avoid the measurement of spurious synchronization between neural sources, by unmixing the summed neural signals recorded at the electrodes, even though simulated original signals are not fully recovered by some linear techniques [60]. Nonetheless, there are limitations to such techniques, and it is possible that important neural sources were not discovered in our analysis, that the sources we did discover were somewhat mislocalized (always a problem with EEG, canonical electrode localization, and average brain), or that the inferred signals generated by these sources contained some mixture of signals from other brain regions. Convergence of our results with previous studies indicates that these possible errors were not severe, but of course further research, and convergence with additional results, will help to provide a more complete picture.

Second, although the methods used in this report to analyze synchronization have only become available to the neuroscience community in the past 10 years or so (e.g., [61]), additional methods have been developed by physicists in the same time frame and have been applied to chaotic and other complex systems, including a few in neuroscience (e.g., [11], [12]). These methods, such as recurrence analysis, can provide a more detailed description of the various regimes of stochastic synchronization and their transitions in complex systems. In particular, information-based measures of synchronization can reveal non-linear relationships between the time courses of complex oscillators, and can even reveal directionality of influence in their time series (e.g., [62]). Nonetheless, time-frequency plots of phase-locking statistics based on signal phases derived from either wavelet analysis or analytic signal construction for narrow-band signals has been shown in numerous studies to provide a reasonable first pass at describing the dynamics of synchronization for both EEG and MEG recordings. Indeed in some cases rather complete descriptions of the oscillatory dynamics of relatively simple brain systems, e.g., that involved in Parkinsonian tremor, have been achieved by such techniques [63]. For this reason we limited our analyses in the present study to such techniques.

The present experiment has provided new evidence that adding small amounts of random variation to a weak stimulus can enhance the brain's response to that stimulus relative to that response without the added noise. The nature of the response recorded here, the 40-Hz transient auditory response, is such that the noise must have enhanced the synchronization of the 40-Hz oscillations of the neurons tuned to the stimulus frequency. This occurred both for standards mixed with noise and standards presented with noise in the opposite ear, in the latter case with noise and stimulus activity mixed in the brain. Moreover, cross-coherence (phase-locking) between the brain regions displaying an enhanced 40-Hz response was also affected by the added noise, with more synchronization occurring in alpha and gamma bands in added noise conditions, often within the 0–100 ms 40-Hz response window. Both of these results confirm the prediction that, as occurs in simulations of model spiking neural networks, random neural noise, in this case created by adding acoustical noise to a sensory receptor, can enhance neural synchronization in a functionally relevant way. These and earlier results indicate that stochastic resonance could play an important role in the transient formation and dissolution of networks of brain regions that underlie perception, cognition, and action. Endogenous noise levels fluctuate widely in the brain over the sleep-wake cycle and within its different phases, as well as with environmental demands, mostly determined by activity in the reticular activating system and the more specific arousal system mediated by the thalamus [64]–[65]. If neural network formation is at least partially governed by the prevailing level of neural noise, it is possible that SR plays an important role in communication within and between brain regions, as the oscillatory synchronization that facilitates that communication is modulated by the prevailing endogenous noise level.

## Methods

### Subjects

Twelve right-handed volunteers (8 men) attending UBC, aged 18–33 years, were paid to participate. All provided written consent. The experiment was approved by the Behavioural Research Ethics Board of the University of British Columbia. All participants were assessed by clinical audiometry and found to have hearing within normal range at the time of the EEG acquisition. No history of neurological disorders was reported during a prescreening interview. Data from two subjects were excluded from the analysis reported here, one because of an error in data collection and the other because their data failed to yield usable ICs in any of the four clusters we studied intensively, leaving 10 subjects (3 women) with usable data.

### Stimuli and procedure

Subjects were seated alone in a sound attenuated chamber throughout the experiment. Stimuli consisted of 5 dB SL (Sensation Level  =  dB above 50% absolute threshold), 60-ms-duration, standard pure tones at 1 kHz and 0.5 kHz presented in an alternating fashion to left and right ears respectively through high-quality insert earphones (E-A-RTONE 3A 10 Ohm; 45 dB minimum interaural attenuation, 25 dB minimum ambient noise attenuation), replaced randomly with occasional (5%) deviant tones at 20 dB SL at each frequency. Broadband auditory noise (125 Hz to 10 kHz) was presented continuously to the left ear (which also received the 1 kHz tones) at six different levels: no-noise, and -5, 0, 5, 10 and 20 dBA SL. Noise conditions were run in separate blocks of 500 stimuli in each ear (475 standards, 25 deviants) twice in counter-balanced orders across subjects, for a total of 950 standards and 50 deviants per noise condition per subject. Subjects were required to press a button on a keyboard each time they heard a deviant tone *in the left ear only* (e.g., in the 1 kHz tone), so that they were attending to the stimulus stream (1 kHz tones plus noise) in the left ear and ignoring that in the right ear (0.5 kHz tones only). Absolute thresholds were acquired separately for 1 kHz and 0.5 kHz pure tones and broadband noise using a 1-up 1-down adaptive staircase before any of the noise conditions were run. This procedure yielded a 50% absolute threshold; subjects reported that the 5 dB SL standards were often inaudible in the no-noise condition. The 20 dB SL noise rendered all standards in the left ear inaudible but deviants were still detected in that condition as in the others. The left ear noise had little effect on the audibility of the right ear standards or deviants as contralateral masking is very weak and inter-ear attenuation by the insert earphones was 45 dB or greater at all frequencies. Performance on the deviant detection task was nearly perfect for all subjects, with less than 1% errors for any subject.

### EEG recording

Data were collected from 60 scalp electrodes mounted in a standard electrode cap (Electrocap, Inc.) at locations based on the International 10-10 System, and from four periocular electrodes placed above and below the right eye and at the right and left outer canthi. During recording all scalp channels were referenced to the right mastoid. Electrode impedance was kept below 20 kΩ for all scalp electrodes (sufficient because SA amplifier input impedence was greater than 2 gΩ). Data were sampled at 500 Hz through an analog passband of 0.01–100 Hz. Prior to analysis, all signals were re-referenced to an average reference to give equal weight to each electrode, then resampled to 250 Hz, and digitally high-pass filtered at 1 Hz. The continuous EEG data were analyzed with EEGLAB software [66], an open source MATLAB (Mathworks, Natick, USA) toolbox available at http://sccn.ucsd.edu/eeglab.

### ICA analysis

We decomposed the continuous data from all conditions (twelve 500-trial blocks per subject) with extended infomax ICA directly. Continuous data provide ample observations, required by ICA, to separate two or more independent neural processes. We used the EEGLAB *runica* algorithm, which is based on the infomax neural network algorithm [67], an algorithm that exploits temporal informational independence to perform blind separation. The 64-channels by time matrix of EEG data, **X**, was transformed into a matrix of 64 independent component activations by time, **U**, by premultiplying **X** by a weight matrix, **W**, of unmixing coefficients, **U = WX**. **W** was derived iteratively to yield 64 non-Gaussian activity sources that were as nearly informationally independent relative to one another as possible [43]–[44], [68].

Once the ICs were calculated, a scalp map for each IC was computed from the inverse of the weight matrix, ***W^−1^***, giving the relative strength of the IC at each electrode averaged over time. This scalp map was then compared with the forward solutions for various single equivalent dipoles. The digitized canonical 10-10 system 3-D locations of the 60 scalp electrodes were first co-registered with the Montreal Neurological Institute (MNI) average brain. IC sources were then localized using the dipfit2 algorithm in EEGLAB using a boundary element model. This algorithm estimates the location and orientation of an equivalent dipolar source for a given scalp potential distribution by a gradient descent method. Only ICs with scalp maps having an inverse solution for a single dipole source within Talairach space (within the brain) with less than 15% residual variance (RV) were included in the subsequent analyses; these were termed “valid ICs.” Those sourced outside the head or with higher RVs were rejected as artifactual or uninterpretable.

Wavelet coefficients of the sinusoidal oscillations in each of several (logarithmically increasing width) frequency bands between 5 and 55 Hz were obtained from a Morlet wavelet analysis (EEGLAB) performed on the broadband activation of each IC. Event-related spectral perturbations (ERSPs) were computed from the wavelet-coefficient-derived spectral power at each time point in each frequency band, relative to the average in the baseline time window from −150 ms to −50 ms in that frequency band, and expressed in dB.

The continuous record of each valid IC was divided into six groups of epochs for standard trials (approximately 950 trials/noise condition/subject), one for each noise condition. Extracted epochs ranged from −150 ms to +450 ms relative to stimulus onset. The time period from −150 ms to −50 ms was considered to be the baseline, and the time period from 0 ms to 100 ms was the 40-Hz transient response window. We also extracted similar epochs around the deviant stimuli (about 50 trials/noise condition/subject) in order to examine them for the precise frequency range of the 40-Hz response to audible stimuli, as this varies somewhat across individuals.

To determine which neural sources were common to the group of subjects, a cluster analysis of all valid ICs was performed based on the dipole locations alone. A total of 200 valid ICs for the 10 subjects were separated into 20 clusters by applying the *k*-means algorithm of EEGLAB. This algorithm attempts to find the centers of natural clusters in the data by minimizing the total intra-cluster variance, or the squared error function. A drawback of the algorithm is that it has to be told the number of clusters (i.e. *k*) to find. We decided upon 20 clusters because that number yielded tight clusters containing most of the subjects in brain regions likely to be relevant to the 40-Hz transient response to the standards, in particular the two primary auditory regions in left and right superior temporal gyri, as well as two other likely-to-be-relevant locations. Greater or lesser numbers of clusters yielded the same four principle clusters.

Normalized total spectral power relevant to the 40-Hz transient response for each cluster-selected IC for each subject was obtained by summing the ERSPs for each time point and each frequency band across a time-frequency window. The time window was fixed at a conventional 0 ms to 100 ms after stimulus onset. The relevant frequency band was determined in two ways: (1) *broad* from 30 Hz to 50 Hz [35], and (2) *custom*, in which the frequency range for each subject was adjusted to that displayed by the 40-Hz transient response to the readily audible deviants, if available, or if not to 35 Hz to 45 Hz. Results were strongest for the custom range for left standard responses and for the broad range for right standard responses. The summed ERSPs were exponentiated to convert them to power ratios and then normalized by dividing by the maximum power ratio across the six noise conditions. Thus, normalized spectral power ratio ranged from near 0 to 1. Normalization was necessary because different subjects had peak power ratio at different noise levels, as is common in such studies [29], [30].

Cross-coherences (phase locking values) were computed from the time series of phases of the sinusoidal oscillations determined by the wavelet analysis for each cluster-selected IC, with number of cycles in the wavelet increasing with frequency by a factor of 0.5/band. Cross-coherence is defined as
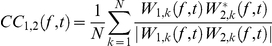
where the *W_i,k_* (*f,t*) are the wavelet coefficients for each time, *t*, and frequency, *f*, point for each IC, *i*, and *k* = 1 to *N* is the index of trials [67]. Cross-coherence, or phase locking, values range from 0 (indicating no phase locking) to 1 (indicating perfect phase locking). Perfect phase locking does not occur with natural (noisy) stimuli; rather a form of stochastic phase locking is commonly observed between naturally-running noisy oscillators such as networks of neurons, in which phase differences remain bounded within a certain relatively small interval although varying across that interval over time or trials (see [7] for a discussion). This analysis was done for frequency bands from 5 to 55 Hz that increased in width logarithmically with frequency.

We required that two different criteria be met in order to conclude that we had observed SR effects in phase locking between brain regions. First, we required that phase locking values in at least some added noise conditions be significantly different from those in the no-noise condition; this is the classical test for SR. To test this we performed a non-parametric permutation-resampling test [68] between the added noise condition and the no-noise condition for each of the time-frequency windows of interest (indicated in Figure 3). For each time-frequency window we took *all* of the available cross-coherence values in the two conditions to be compared (that is, all of the values in that window for those subjects who had an IC in each of the relevant clusters), randomly assigned them to one or the other of two arbitrary groups, and calculated the average cross-coherence difference between these “pseudo-conditions.” This was done 1000 times to create a surrogate distribution of differences. The actual average cross-coherence difference between the added-noise condition and the no-noise condition was then compared to this surrogate distribution and if it was greater than *all* of the 1000 surrogate differences it was considered to be significant at *p*<0.001. This procedure has been shown to control for experiment-wise Type I error in the Bonferroni sense [69]. Significant differences by this test were considered to have met the first criterion and thus to be candidates for entry into Figure 3.

Not all such significant differences were considered to be generalizable SR effects, however. Because of the nature of the permutation test, which included a large group of phase locking values, a few subjects with very large differences could dominate the overall average difference. Moreover, it is possible that the permutation test could show a significant result even if none or only a few subjects had individual cross-coherences that were significantly different from zero. A difference between the no-noise and an added-noise condition in which most subjects' phase locking values did not differ from zero in the added-noise condition would be meaningless. Thus, as a second criterion, we used the EEGLAB procedure for determining whether a group of cross-coherences is generally different from zero to filter the values we entered into Figure 3. In this procedure, individual subjects' cross-coherences were masked at *p*<0.01 for each of a number of smaller time-frequency windows (the grain of the wavelet analysis, hereafter called “pixels”) within each larger time-frequency window in each condition separately, and the group of masked coherences was masked at *p*<0.001 or less. Masking for individual coherences in each pixel was done with a permutation (surrogate) method based on 200 shufflings of the epochs for each IC involved, and that for the group was done with a binomial probability calculation. In the latter case, the *p*-value used for the individual tests was taken as the probability of a “success” in a binomial distribution with *P(failure)* = 1-*P(success*), and the binomial probability of *k* or more of *n* individuals with a significant coherence at *p*<0.01 was kept less than 0.001 (the minimum binomial probability was determined by the number of individual IC pairs available). Figure S2 shows the results of these tests for each pair and each condition. We entered into Figure 3 only those significant differences between the no-noise and an added-noise condition (from the first test) in which a cluster of pixels in the relevant added-noise condition was significantly different from zero cross-coherence within the indicated time-frequency window (see the indicated panel of Figure S2; from the second test), meaning that all or most of the subjects had significantly greater than zero cross-coherence for those pixels. An exception was for the theta band for RSTG-LSFG and RSTG-LPCi pairs, where cross-coherence was consistently different from zero for many pixels in nearly all noise conditions, whether they differed between no-noise and added-noise conditions or not (see Figure 3 and Figure S2).

## Supporting Information

Figure S1 Power ratio results for left standard 30–50 Hz window and right standard custom window.
(0.94 MB PDF)

Figure S2 Time-frequency graphs of cross-coherences for each pair of ICs, masked at binomial p<0.001, used to filter entries in Figure 3.
(6.59 MB PDF)
