# Supplementary material for: Stochastic Resonance Modulates Neural Synchronization within and between Cortical Sources
Source: PLoS One. 2010 Dec 16;5(12):e14371. doi: 10.1371/journal.pone.0014371 (PMC3002936; doi:10.1371/journal.pone.0014371)
Supplement: Figure S1 — Power ratio results for left standard 30–50 Hz window and right standard custom window. (0.94 MB PDF) [file pone.0014371.s001.pdf]

Figure S1 displays the normalized power ratios for the four brain regions of interest in separate labeled panels as a function of the noise level for the Left Standard 30-50 Hz frequency window (top) and for the Right Standard Custom frequency window (bottom). Figure 2 of the main text displays the complementary data, Left Standard custom window and Right Standard 30-50 Hz window.

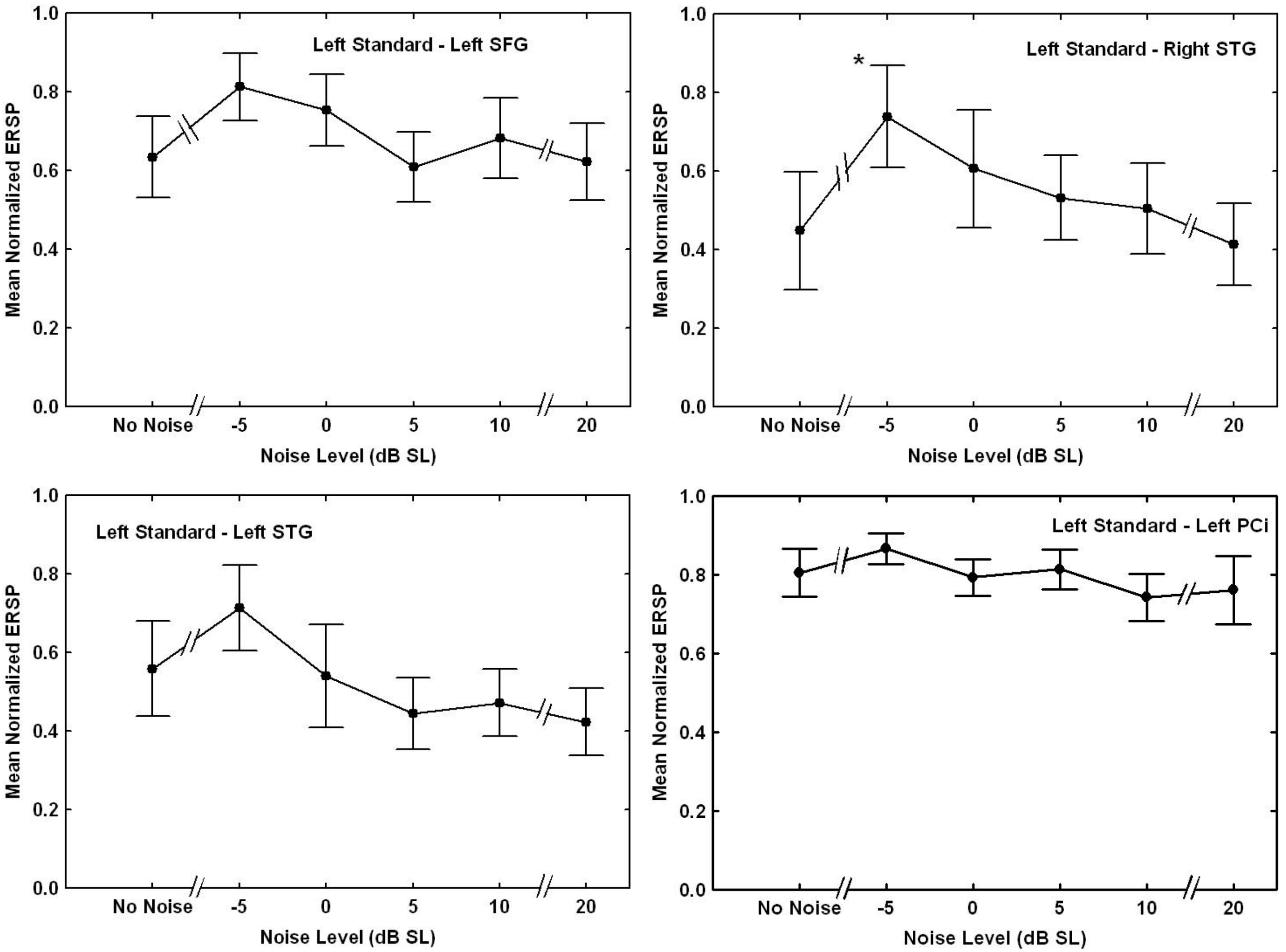


Left Standards 30-50 Hz Window

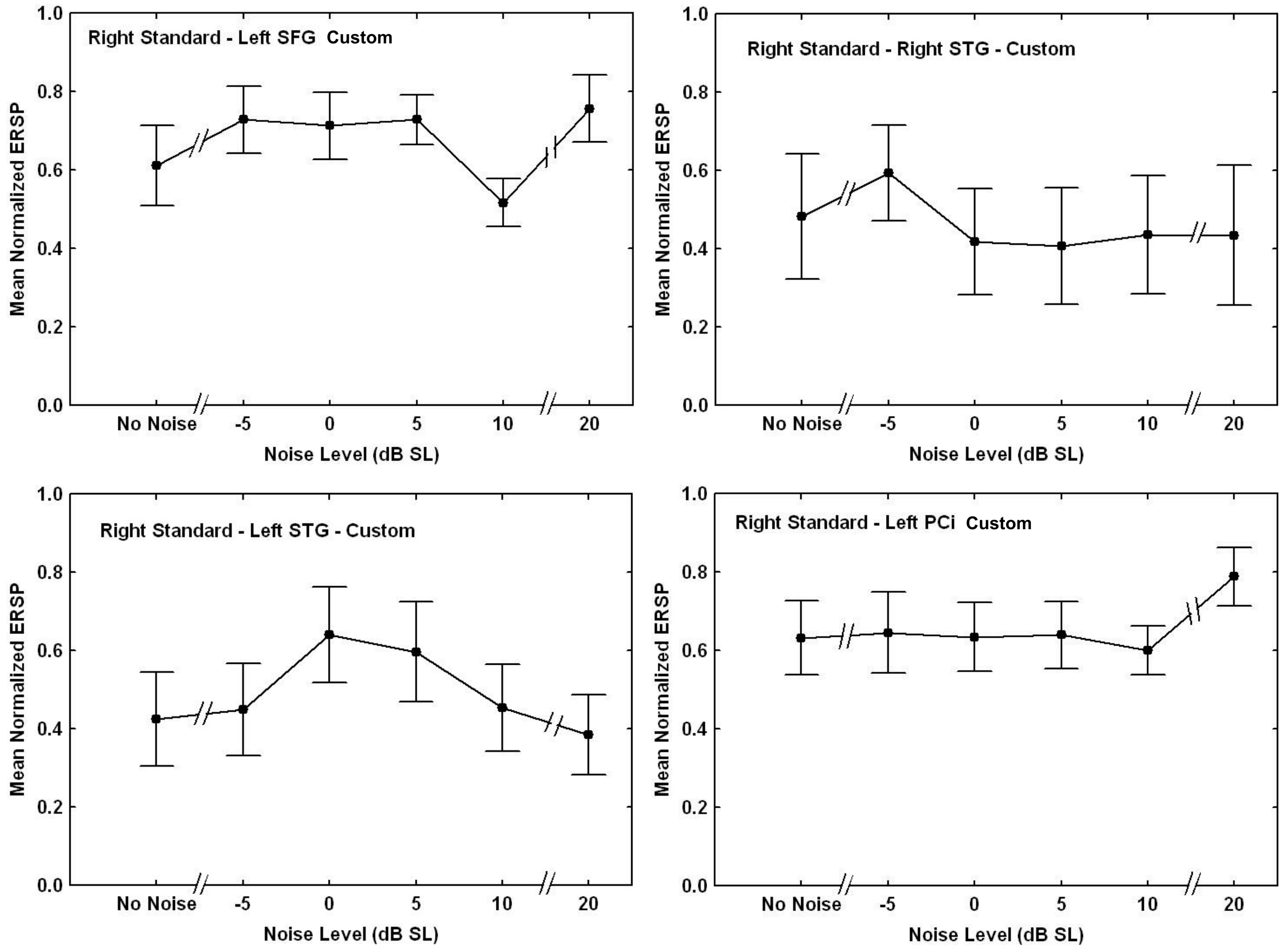


Right Standards Custom Window
