# Supplementary material for: Stochastic Resonance Modulates Neural Synchronization within and between Cortical Sources
Source: PLoS One. 2010 Dec 16;5(12):e14371. doi: 10.1371/journal.pone.0014371 (PMC3002936; doi:10.1371/journal.pone.0014371)
Supplement: Figure S2 — Time-frequency graphs of cross-coherences for each pair of ICs, masked at binomial p<0.001, used to filter entries in Figure 3. (6.59 MB PDF) [file pone.0014371.s002.pdf]

Each panel of Figure S2 indicates the source pair involved and displays a time-frequency graph of the masked cross-coherence values (phase-locking values) for that pair. The scale at the right of each figure indicates the values of these statistics, and the non-zero values displayed are the means of the cross-coherences calculated across the individual subjects who had ICs in both of the relevant clusters and whose phase locking values were significantly greater than 0 ($p<0.01$ by surrogate analysis). Mean cross-coherence values were masked at the binomial p-values (probability of Type I error) indicated on each figure for each pixel with binomial P(success)=0.01 and P(failure)=1-P(success)=0.99 from surrogates run on individual subjects' cross-coherence matrices (EEGLAB/MatLab script). Minimum binomial p-values are inversely related to the number of subjects who had an IC in both members of the particular pair of source clusters. Note that "significant" pixels in these figures indicate that all (when fewer subjects had an IC in both indicated clusters) or most (when more subjects had ICs in both clusters) had cross-coherences greater than 0 by the surrogate technique applied to individual subjects' data. These *do not constitute statistical contrasts* between any of the conditions; each graph in a given panel displays masked averages for only a single condition. Figure 3 in the main paper indicates which added noise conditions showed a statistically significant difference ($p<0.001$) between the added noise condition and the zero-noise condition using the permutation test described there (based on *all* of the subjects' cross-coherences in that time-frequency window, not just the ones showing significantly non-zero cross-coherences) *and also* showed a cluster of pixels within the indicated time-frequency window in the indicated one of the figures shown here meaning that all or most of the subjects had significantly greater than zero cross-coherence for those pixels. If the statistical contrast was significant but the majority of the subjects did not show significantly non-zero cross-coherence in the added noise condition at least, that square of Figure 3 is colored gray, as the result of the permutation test in that instance might have arisen from a large difference shown by only a few subjects. Conversely, if the statistical contrast was not significant, regardless of whether any added noise condition had a cluster of pixels with non-zero cross-coherence in an added-noise condition, that square is also gray in Figure 3. The only exception is for the theta band for the RTSG-LSFG and RTSG-LCPi comparisons, where most subjects had significantly non-zero cross-coherences for all noise conditions.

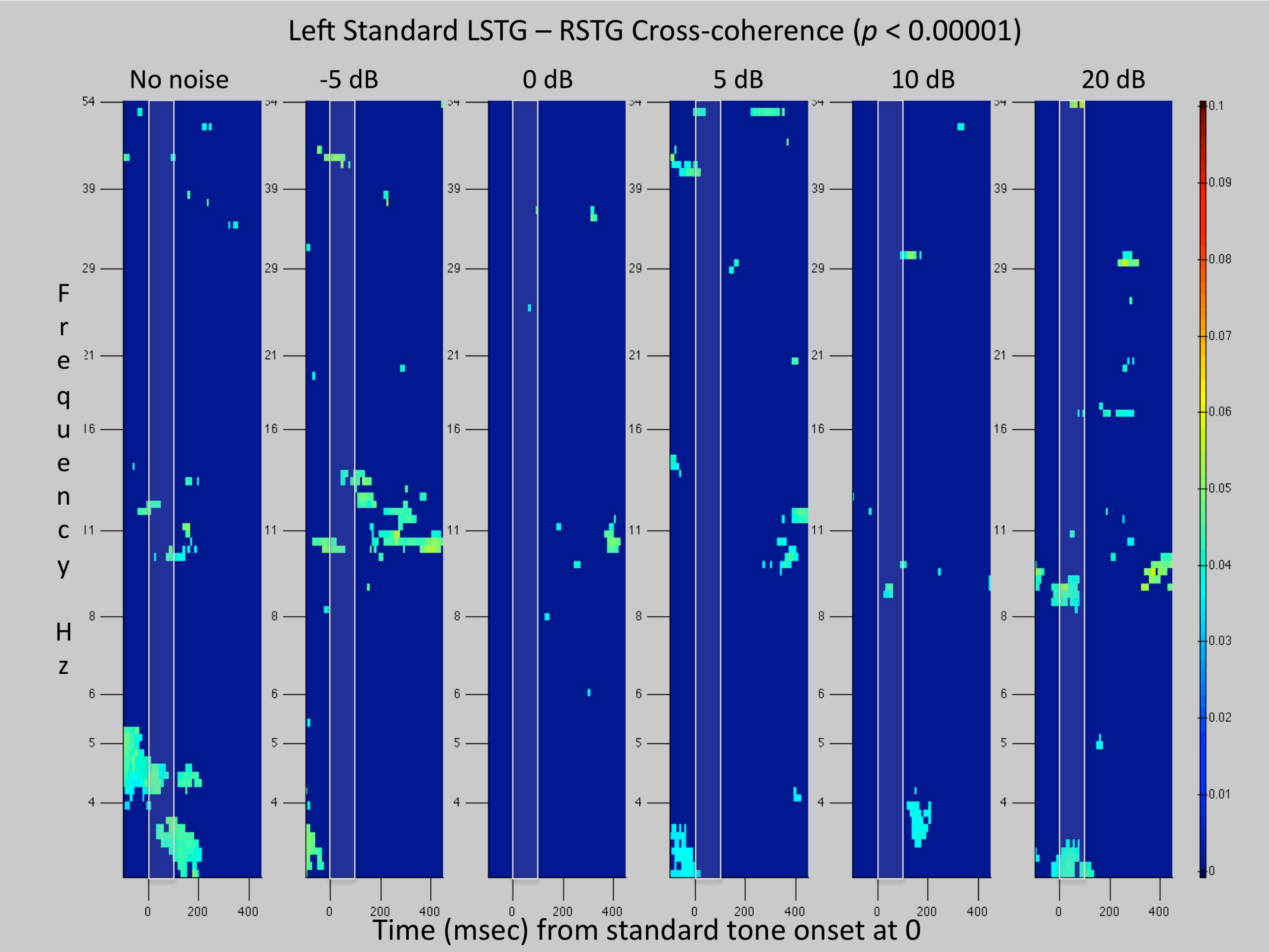

Left Standard LSTG – RSTG Cross-coherence (p < 0.00001)
No noise
-5 dB
0 dB
5 dB
10 dB
20 dB
Frequency Hz
54
39
29
21
16
11
8
6
5
4
0
200
400
0.1
0.09
0.08
0.07
0.06
0.05
0.04
0.03
0.02
0.01
0
Time (msec) from standard tone onset at 0

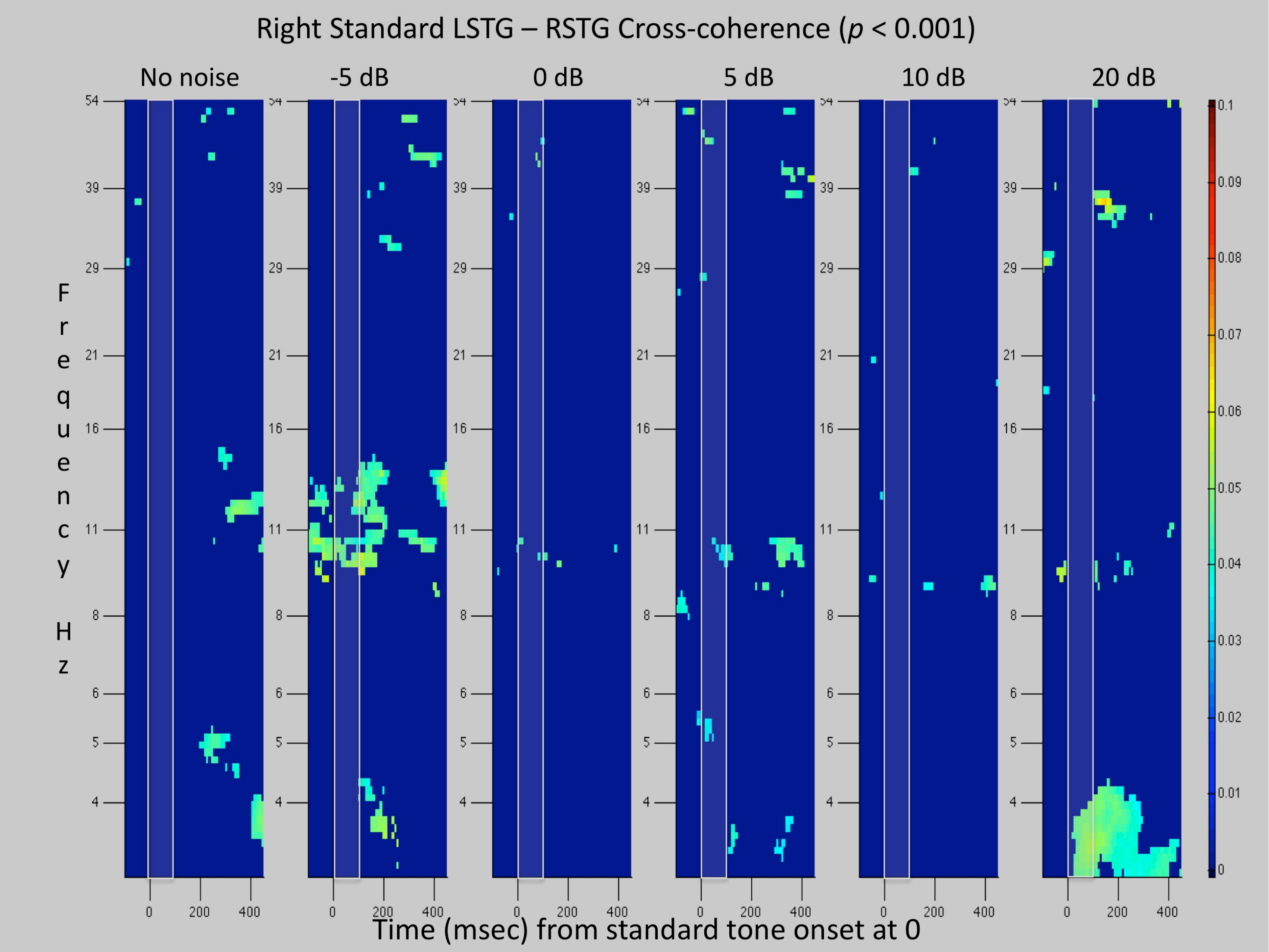
Right Standard LSTG – RSTG Cross-coherence (p < 0.001)
No noise
-5 dB
0 dB
5 dB
10 dB
20 dB
Frequency Hz
54
39
29
21
16
11
8
6
5
4
0
200
400
0.1
0.09
0.08
0.07
0.06
0.05
0.04
0.03
0.02
0.01
0
Time (msec) from standard tone onset at 0

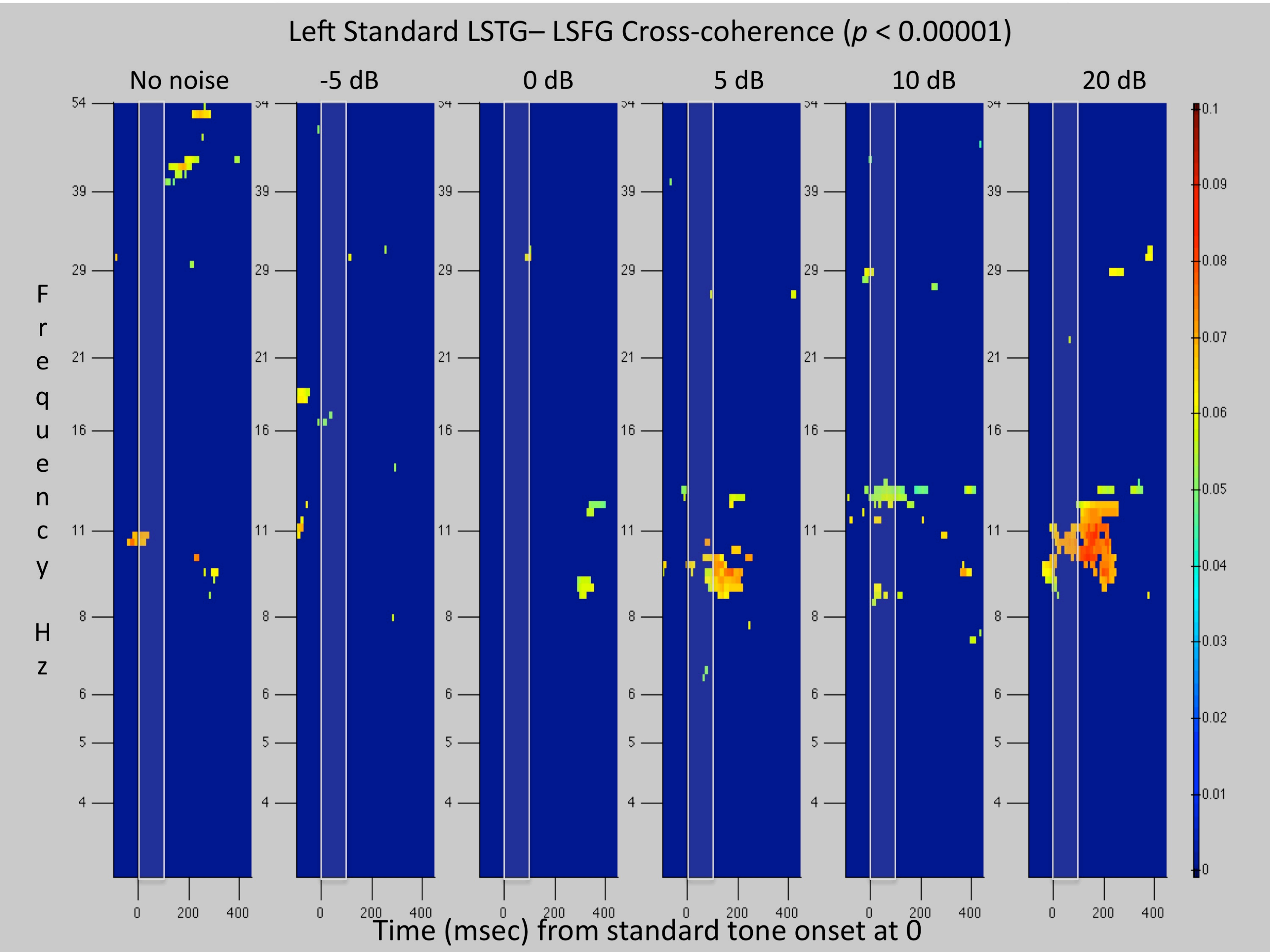

Left Standard LSTG– LSFG Cross-coherence (p < 0.00001)
No noise
-5 dB
0 dB
5 dB
10 dB
20 dB
Frequency Hz
54
39
29
21
16
11
8
6
5
4
0
200
400
0.1
0.09
0.08
0.07
0.06
0.05
0.04
0.03
0.02
0.01
0
Time (msec) from standard tone onset at 0

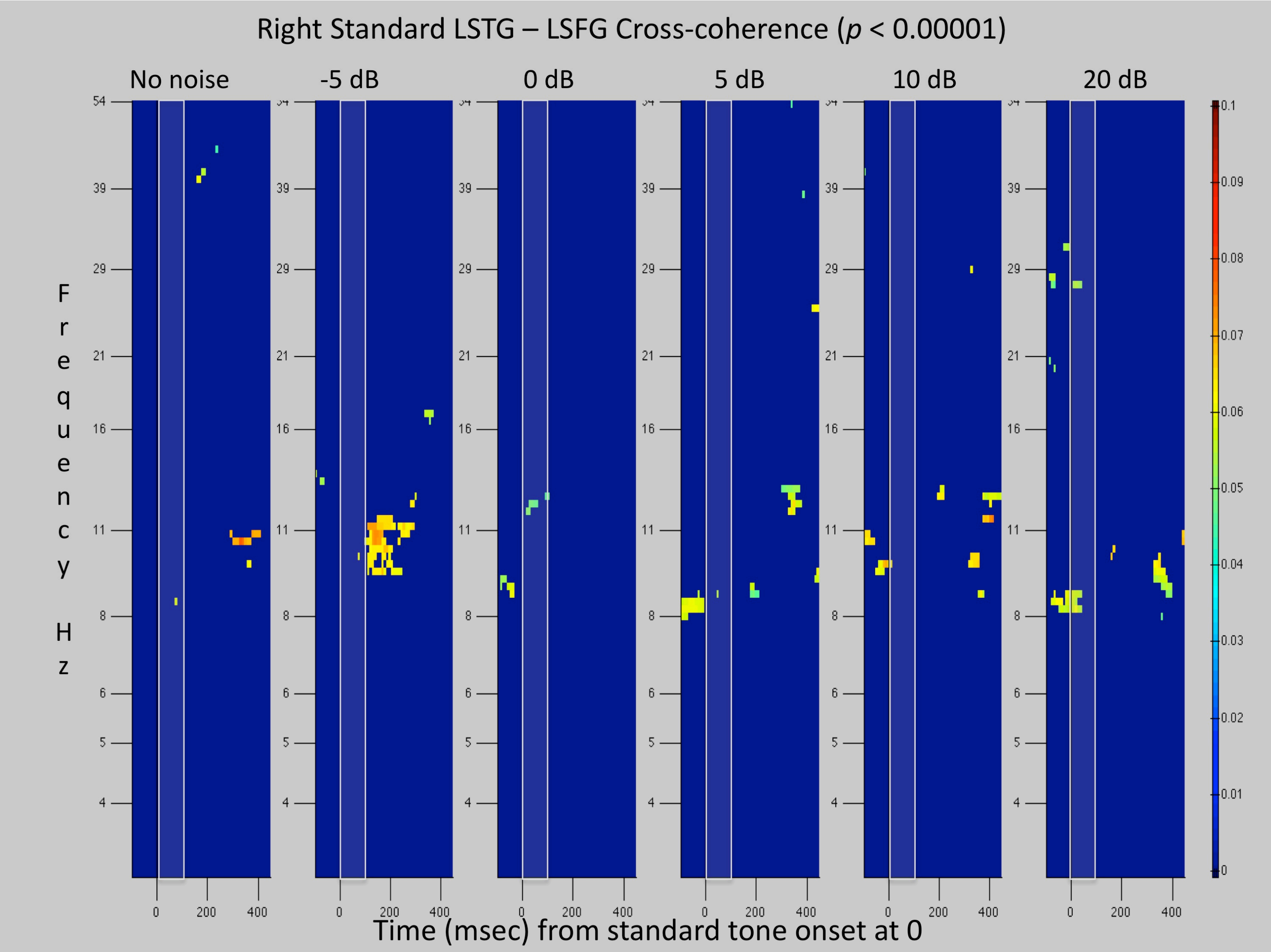

Right Standard LSTG – LSFG Cross-coherence (p < 0.00001)
No noise
-5 dB
0 dB
5 dB
10 dB
20 dB
Frequency Hz
54
39
29
21
16
11
8
6
5
4
0
200
400
0.1
0.09
0.08
0.07
0.06
0.05
0.04
0.03
0.02
0.01
0
Time (msec) from standard tone onset at 0

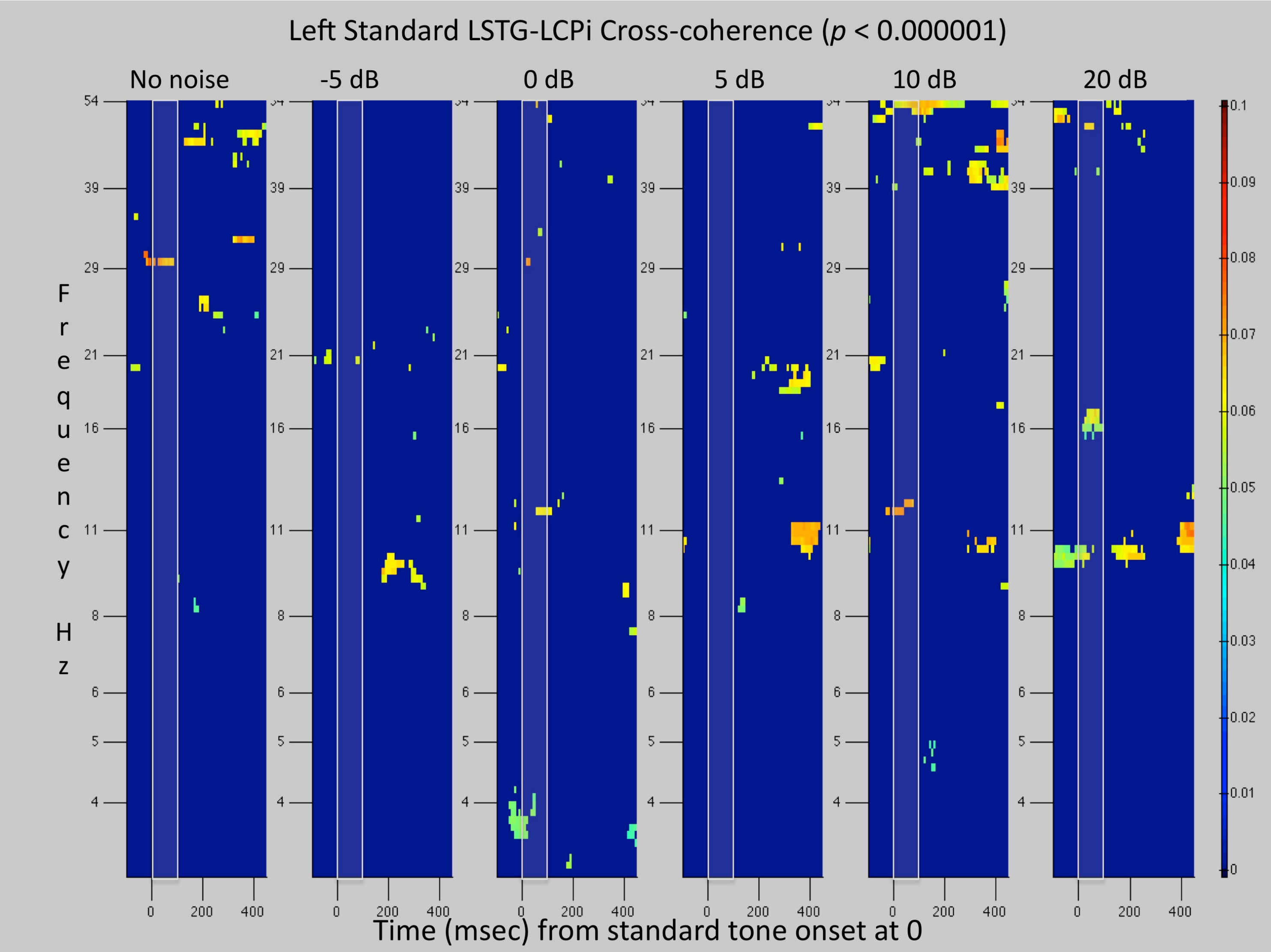

Left Standard LSTG-LCPi Cross-coherence (p < 0.000001)
No noise
-5 dB
0 dB
5 dB
10 dB
20 dB
Frequency Hz
54
39
29
21
16
11
8
6
5
4
0
200
400
0.1
0.09
0.08
0.07
0.06
0.05
0.04
0.03
0.02
0.01
0
Time (msec) from standard tone onset at 0

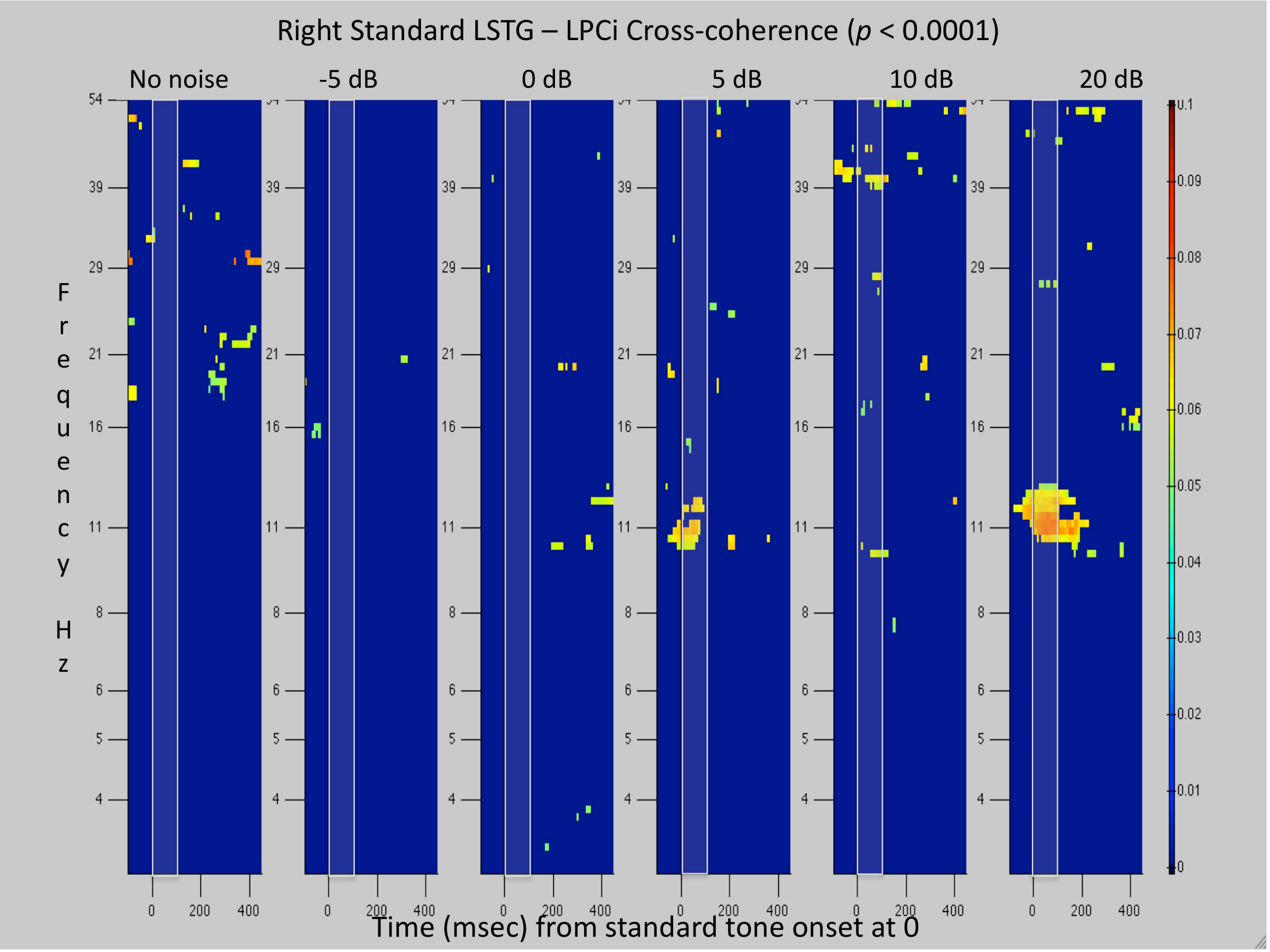

Right Standard LSTG – LPCi Cross-coherence (p < 0.0001)
No noise
-5 dB
0 dB
5 dB
10 dB
20 dB
Frequency Hz
54
39
29
21
16
11
8
6
5
4
0
200
400
0.1
0.09
0.08
0.07
0.06
0.05
0.04
0.03
0.02
0.01
0
Time (msec) from standard tone onset at 0

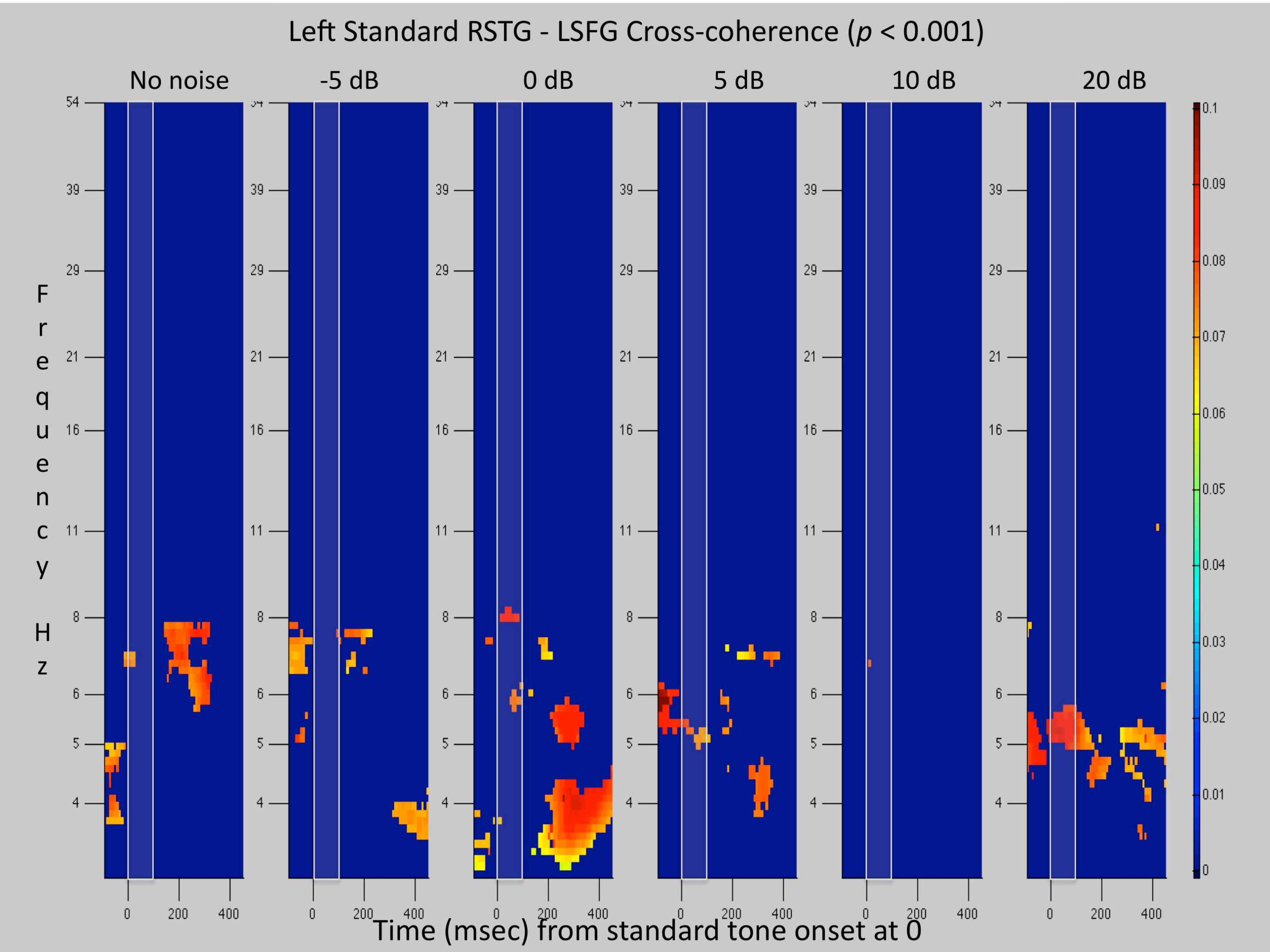

Left Standard RSTG - LSFG Cross-coherence (p < 0.001)
No noise
-5 dB
0 dB
5 dB
10 dB
20 dB
Frequency Hz
54
39
29
21
16
11
8
6
5
4
0
200
400
0.1
0.09
0.08
0.07
0.06
0.05
0.04
0.03
0.02
0.01
0
Time (msec) from standard tone onset at 0

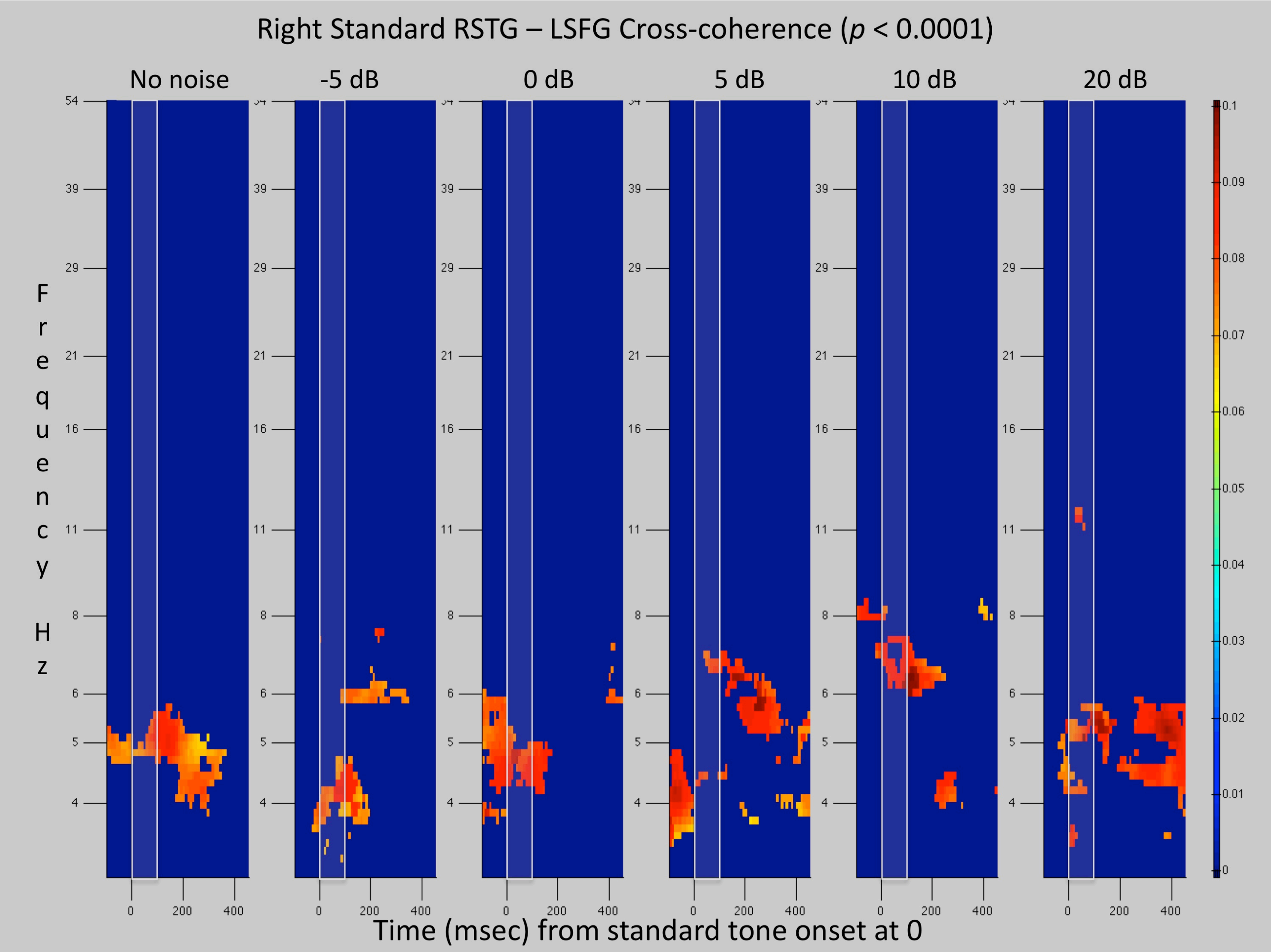
Right Standard RSTG – LSFG Cross-coherence (p < 0.0001)
No noise
-5 dB
0 dB
5 dB
10 dB
20 dB
Frequency Hz
54
39
29
21
16
11
8
6
5
4
0
200
400
0.1
0.09
0.08
0.07
0.06
0.05
0.04
0.03
0.02
0.01
0
Time (msec) from standard tone onset at 0

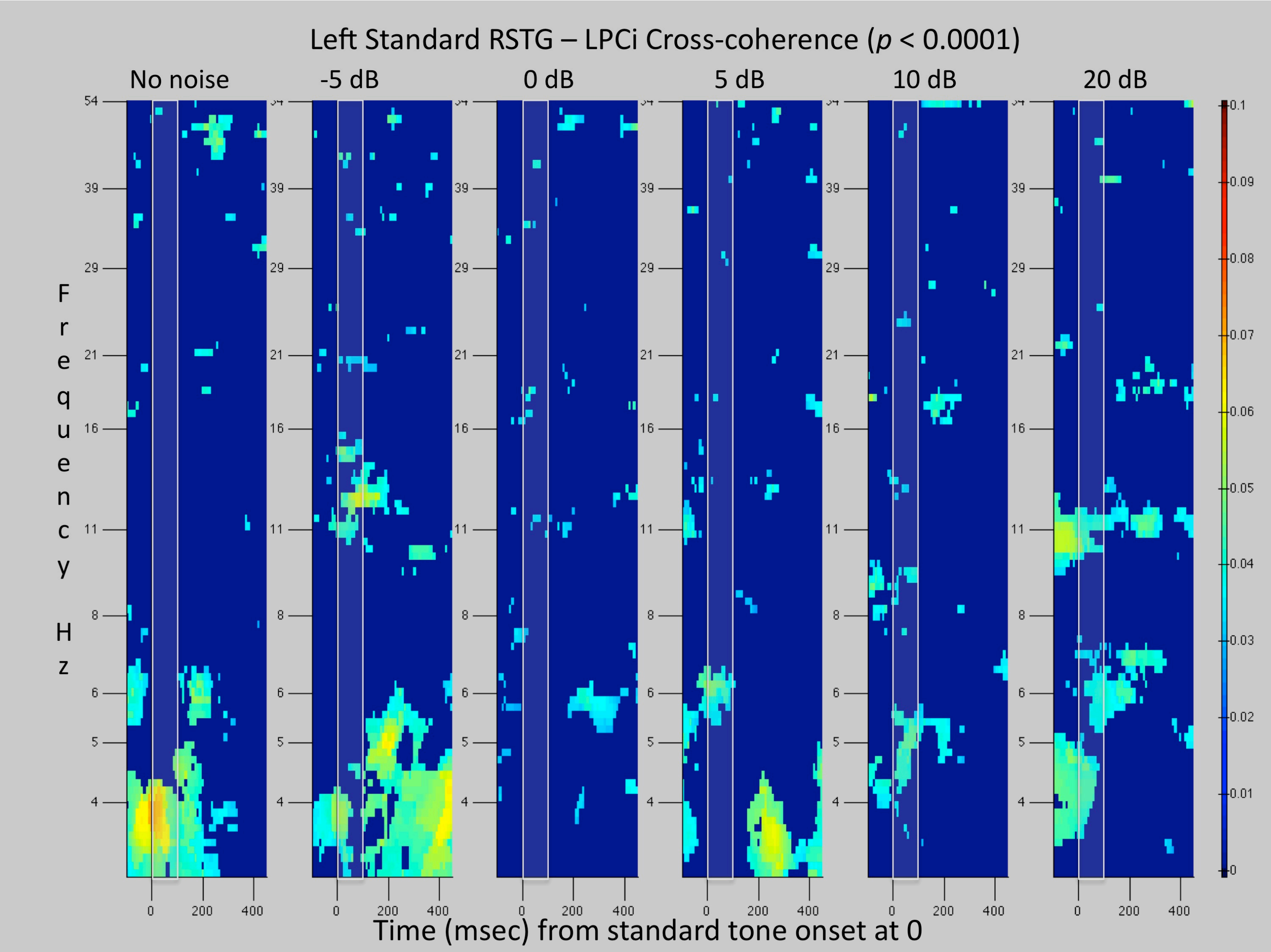

Left Standard RSTG – LPCi Cross-coherence (p < 0.0001)
No noise
-5 dB
0 dB
5 dB
10 dB
20 dB
Frequency Hz
54
39
29
21
16
11
8
6
5
4
0
200
400
0.1
0.09
0.08
0.07
0.06
0.05
0.04
0.03
0.02
0.01
0
Time (msec) from standard tone onset at 0

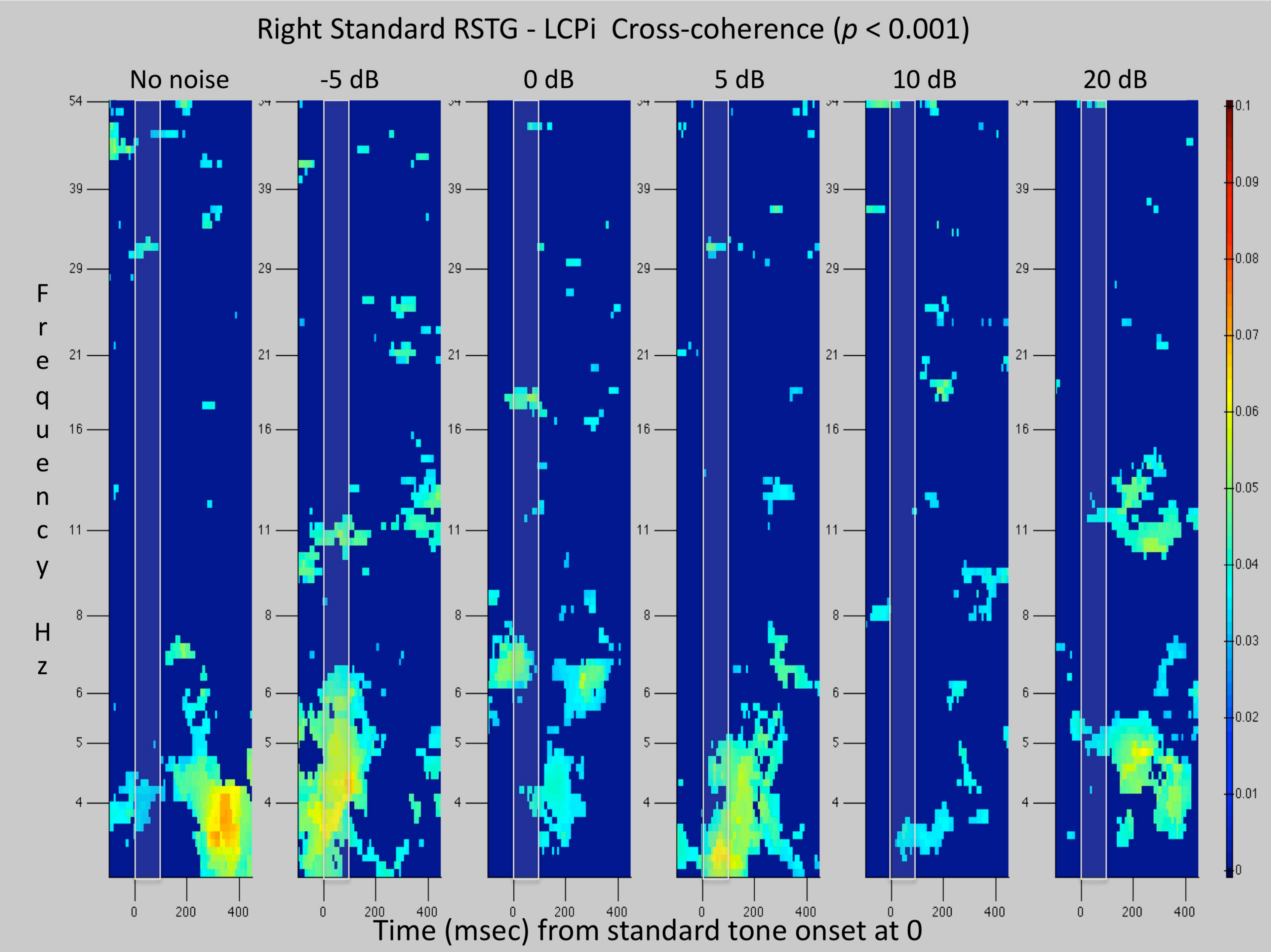
Right Standard RSTG - LCPi Cross-coherence (p < 0.001)
No noise
-5 dB
0 dB
5 dB
10 dB
20 dB
Frequency Hz
54
39
29
21
16
11
8
6
5
4
0
200
400
Time (msec) from standard tone onset at 0
0.1
0.09
0.08
0.07
0.06
0.05
0.04
0.03
0.02
0.01
0

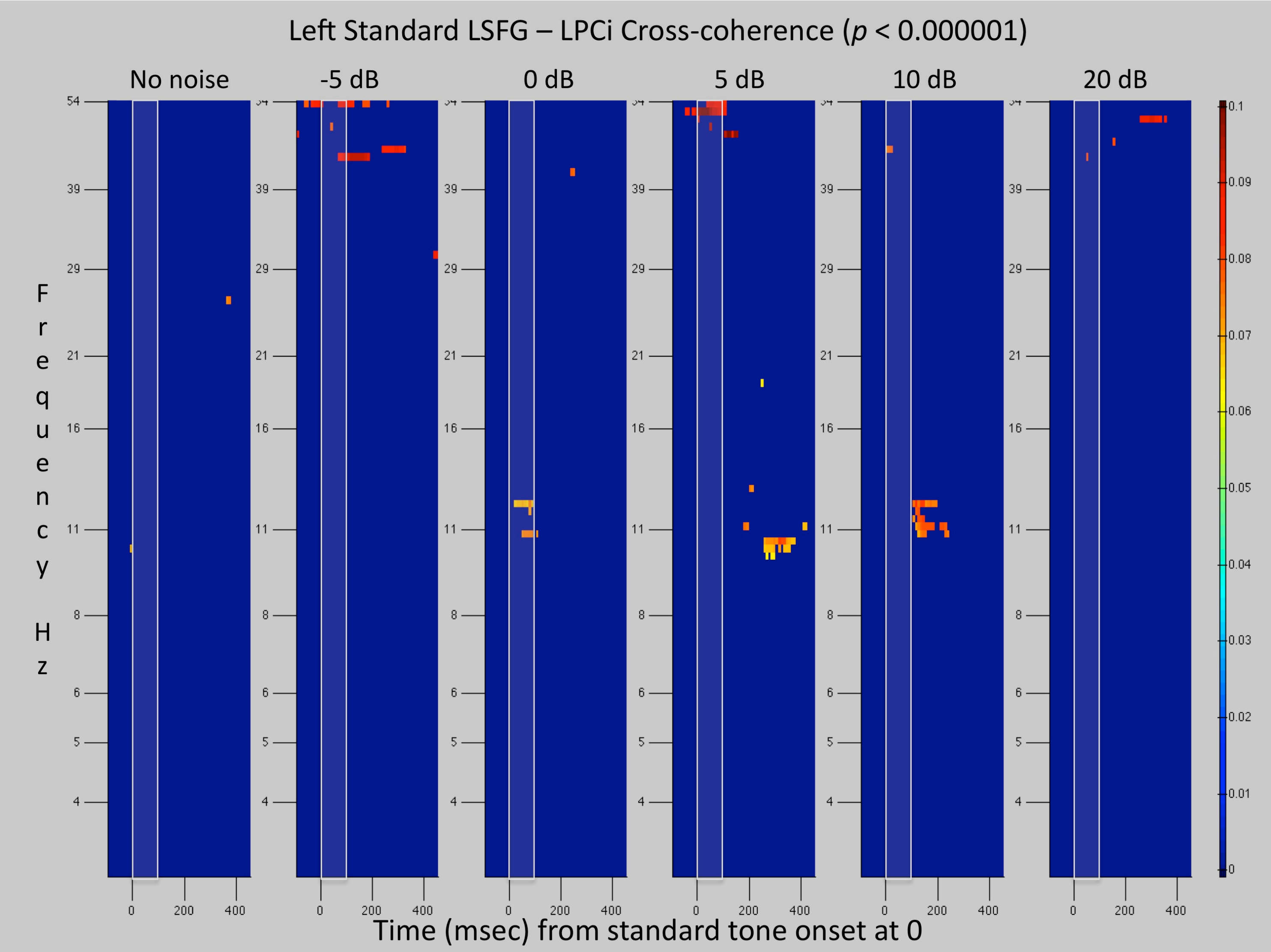

Left Standard LSFG – LPCi Cross-coherence (p < 0.000001)
No noise
-5 dB
0 dB
5 dB
10 dB
20 dB
Frequency Hz
54
39
29
21
16
11
8
6
5
4
0
200
400
0.1
0.09
0.08
0.07
0.06
0.05
0.04
0.03
0.02
0.01
0
Time (msec) from standard tone onset at 0

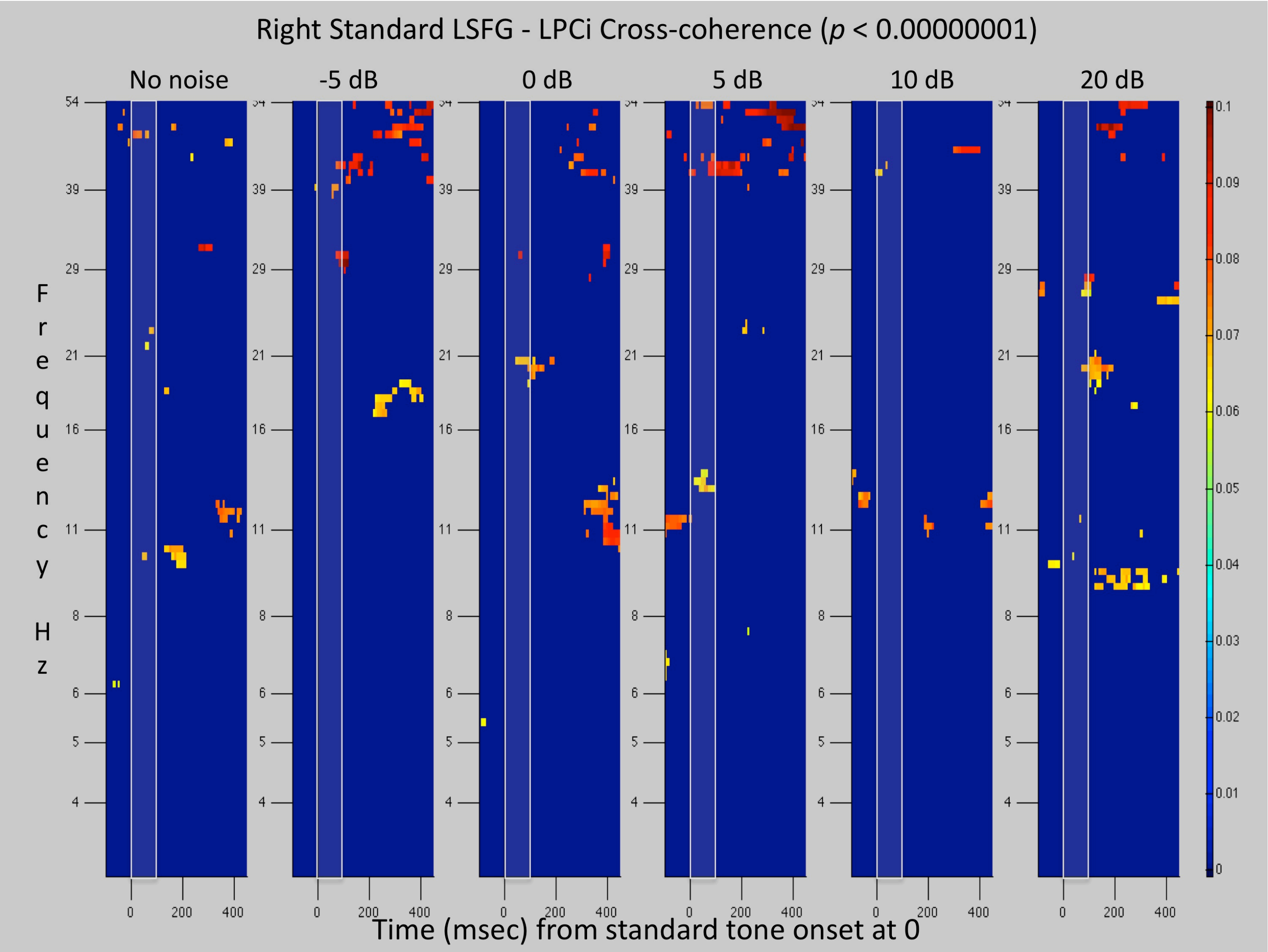
Right Standard LSFG - LPCi Cross-coherence (p < 0.00000001)
No noise
-5 dB
0 dB
5 dB
10 dB
20 dB
Frequency Hz
54
39
29
21
16
11
8
6
5
4
0
200
400
0.1
0.09
0.08
0.07
0.06
0.05
0.04
0.03
0.02
0.01
0
Time (msec) from standard tone onset at 0
